# CD4+ T cells are the major predictor of HCMV control in allogeneic stem cell transplant recipients on letermovir prophylaxis

**DOI:** 10.3389/fimmu.2023.1148841

**Published:** 2023-05-10

**Authors:** Chris David Lauruschkat, Ihsan Muchsin, Alice Rein, Florian Erhard, Denise Grathwohl, Lars Dölken, Carolin Köchel, Christine Susanne Falk, Hermann Einsele, Sebastian Wurster, Götz Ulrich Grigoleit, Sabrina Kraus

**Affiliations:** ^1^ Department of Internal Medicine II, University Hospital of Wuerzburg, Wuerzburg, Germany; ^2^ Institute for Virology and Immunobiology, Julius-Maximilians-University Wuerzburg, Wuerzburg, Germany; ^3^ Helmholtz-Institute for RNA-based Infection Research (HIRI), Helmholtz-Center for Infection Research (HZI), Wuerzburg, Germany; ^4^ Hannover Medical School, Institute of Transplant Immunology, Hanover, Germany; ^5^ TTU-IICH, German Center for Infection Research (DZIF), Hannover-Braunschweig, Germany; ^6^ BREATH Site, German Center for Lung Research (DZL), Hannover-Braunschweig, Germany; ^7^ Department of Infectious Diseases, Infection Control and Employee Health, The University of Texas MD Anderson Cancer Center, Houston, TX, United States; ^8^ Department of Hematology, Oncology and Immunology, Helios Hospital Duisburg, Duisburg, Germany

**Keywords:** human cytomegalovirus (HCMV), viral infection, allogeneic stem cell transplantation, T cells, NK cells

## Abstract

**Introduction:**

Human cytomegalovirus (HCMV) causes significant morbidity and mortality in allogeneic stem cell transplant (alloSCT) recipients. Recently, antiviral letermovir prophylaxis during the first 100 days after alloSCT replaced PCR-guided preemptive therapy as the primary standard of care for HCMV reactivations. Here, we compared NK-cell and T-cell reconstitution in alloSCT recipients receiving preemptive therapy or letermovir prophylaxis in order to identify potential biomarkers predicting prolonged and symptomatic HCMV reactivation.

**Methods:**

To that end, the NK-cell and T-cell repertoire of alloSCT recipients managed with preemptive therapy (n=32) or letermovir prophylaxis (n=24) was characterized by flow cytometry on days +30, +60, +90 and +120 after alloSCT. Additionally, background-corrected HCMV-specific T-helper (CD4+IFNγ+) and cytotoxic (CD8+IFNγ+CD107a+) T cells were quantified after pp65 stimulation.

**Results:**

Compared to preemptive therapy, letermovir prophylaxis prevented HCMV reactivation and decreased HCMV peak viral loads until days +120 and +365. Letermovir prophylaxis resulted in decreased T-cell numbers but increased NK-cell numbers. Interestingly, despite the inhibition of HCMV, we found high numbers of “memory-like” (CD56dimFcεRIγ- and/or CD159c+) NK cells and an expansion of HCMV-specific CD4+ and CD8+ T cells in letermovir recipients. We further compared immunological readouts in patients on letermovir prophylaxis with non/short-term HCMV reactivation (NSTR) and prolonged/symptomatic HCMV reactivation (long-term HCMV reactivation, LTR). Median HCMV-specific CD4+ T-cell frequencies were significantly higher in NSTR patients (day +60, 0.35 % vs. 0.00 % CD4+IFNγ+/CD4+ cells, p=0.018) than in patients with LTR, whereas patients with LTR had significantly higher median regulatory T-cell (Treg) frequencies (day +90, 2.2 % vs. 6.2 % CD4+CD25+CD127dim/CD4+ cells, p=0.019). ROC analysis confirmed low HCMV specific CD4+ (AUC on day +60: 0.813, p=0.019) and high Treg frequencies (AUC on day +90: 0.847, p=0.021) as significant predictors of prolonged and symptomatic HCMV reactivation.

**Discussion:**

Taken together, letermovir prophylaxis delays HCMV reactivation and alters NK- and T-cell reconstitution. High numbers of HCMV-specific CD4+ T cells and low numbers of Tregs seem to be pivotal to suppress post-alloSCT HCMV reactivation during letermovir prophylaxis. Administration of more advanced immunoassays that include Treg signature cytokines might contribute to the identification of patients at high-risk for long-term and symptomatic HCMV reactivation who might benefit from prolonged administration of letermovir.

## Introduction

Allogeneic hematopoietic stem cell transplantation (alloSCT) remains the only curative treatment for many patients suffering from hematologic malignancies (1). However, alloSCT recipients are highly susceptible to opportunistic infections (1–3). Human cytomegalovirus (HCMV) is the most frequent viral complication in alloSCT recipients (4). Upon primary infection, it establishes a life-long latency in its human host; however, a functional immune system is able to efficiently prevent clinically symptomatic HCMV reactivation. In contrast, the delayed reconstitution of the immune system after alloSCT provides a window of opportunity for HCMV reactivation. While some alloSCT patients manage to rapidly control or even prevent HCMV reactivation as measured by weekly quantitative PCR, others develop prolonged and symptomatic HCMV reactivations and potentially end-organ disease (EOD), resulting in high morbidity and mortality (2, 5).

The magnitude, functionality, and specificity of HCMV-specific T- and natural killer (NK)-cell-mediated responses determine the risk and severity of HCMV disease in these patients (4, 6, 7). Delayed or dysfunctional HCMV-directed T-cell responses are the main risk factors for prolonged HCMV viremia and HCMV disease (8, 9). In addition, delayed NK-cell reconstitution also contributes to HCMV reactivation (6, 7). Specifically, so-called “memory-like” NK cells (FcϵRIγ^-^ and/or CD159c^+^), which belong to the CD56^dim^ NK-cell compartment, have been shown to play an important role in preventing or controlling HCMV reactivation and disease (6, 7).

Two main strategies have been adopted to prevent HCMV disease: antiviral prophylaxis and preemptive therapy. Preemptive therapy, that is, monitoring for HCMV reactivation by viral (real-time quantitative) polymerase chain reaction [(q)PCR] performed on a weekly basis and initiation of antiviral treatment upon HCMV detection, used to be the standard of care for HCMV in alloSCT recipients for decades (10, 11). However, the recent approval of letermovir for antiviral prophylaxis has substantially changed the management of HCMV in alloSCT recipients.

Letermovir inhibits the pUL56 subunit of the HCMV terminase complex, thereby preventing cleavage of the concatemeric viral DNA and interfering with HCMV replication. Due to its excellent toxicity profile, its primary prophylactic administration for the first 100 days post-transplantation in seropositive alloSCT recipients is now regularly employed and has led to a significant decrease in virus reactivations and reduced non-relapse mortality (12, 13). Following the introduction of letermovir prophylaxis, early-onset HCMV reactivations have become rare. In contrast, late-onset HCMV reactivations have emerged as a frequent cause of HCMV disease (4). Furthermore, alloSCT patients are still monitored by qPCR for HCMV reactivations. Preemptive therapy is initiated when a certain HCMV load is detected during letermovir prophylaxis. However, it is important to note that while qPCR testing can detect HCMV DNA, it does not necessarily indicate whether the virus is actively replicating or not. Letermovir stops HCMV replication after DNA replication has occurred, which might lead to an abortive infection (14).

The effects of letermovir on T- and (“memory-like”) NK-cell reconstitution has been only partially studied so far (8, 9). Specifically, HCMV-specific T-cell reconstitution kinetics and their impact on transplantation outcomes in patients on letermovir prophylaxis are poorly understood. Here, we compare rates of HCMV reactivation and kinetics of HCMV-specific T- and NK-cell reconstitution in patients receiving PCR-guided preemptive therapy or letermovir prophylaxis in order to identify potentially predictive T- and NK-cell biomarkers for HCMV reactivation and disease.

## Materials and methods

### Institutional review board approval

The study was approved by the Ethics Committees of the University of Wuerzburg (protocol code 17/19-sc). Written informed consent was obtained from all subjects.

### Study population

**Figure 1:**
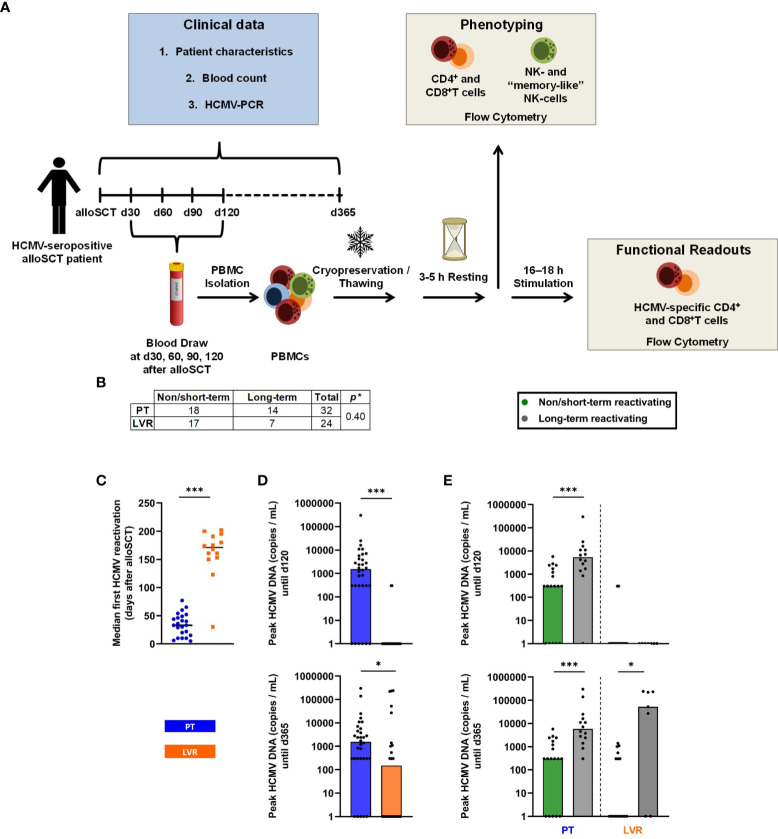
Study design and influence of letermovir prophylaxis on HCMV reactivation. **(A)** Schematic study design. **(B)** Number of patients included in the study, subdivided by treatment and duration of HCMV reactivation (non/short-term HCMV reactivation vs. long-term reactivation). **(C)** 1^st^ median HCMV reactivation in alloSCT recipients receiving letermovir prophylaxis or preemptive therapy. **(D)** Peak HCMV DNA copies/mL measured by PCR until day +120 and day +365 in alloSCT patients receiving letermovir prophylaxis or preemptive therapy. **(E)** Peak HCMV DNA copies/ml measured by PCR until day +120 and day +365 in alloSCT patients receiving letermovir prophylaxis (right) or preemptive therapy (left), with long-term HCMV reactivation (grey) or non/short-term HCMV reactivation (green). **(B-E)** Fisher’s exact test **(B)** or Mann–Whitney *U* test **(C-E)** were used to test for statistical significance: * p < 0.05, *** p < 0.05. Median values are shown (bars and lines). alloSCT, allogeneic stem cell transplantation; CD, cluster of differentiation; d, day; LVR, letermovir; PCR, polymerase chain reaction; PT, preemptive therapy.

Fifty-four adult HCMV-seropositive (Recipient [R]^+^Donor [D]^+^, R^+^D^-^) patients after alloSCT were enrolled at the University Hospital of Wuerzburg from 09/2015 until 11/2021. HCMV DNAemia was quantified by real-time PCR once weekly until day 100 post-transplant and every other week after day +100. PCR-guided preemptive therapy was used as the primary antiviral strategy until 12/2019 (n=32). Preemptive therapy for hospitalized patients was started whenever HCMV DNAemia exceeded 1,000 DNA copies/mL. Patients received a two-week induction therapy with oral/IV (val)ganciclovir, followed by maintenance therapy with the same medication for up to an additional two weeks or until two consecutive negative tests for HCMV viremia were documented. Starting in 1/2020, letermovir prophylaxis was administered to HCMV-seropositive alloSCT recipients (n=22) at a dose of 480 mg letermovir once daily (reduced to 240 mg for patients receiving cyclosporine A), from day +1 to day +100 according to institutional standard practice. Whenever HCMV DNAemia exceeded 1,000 DNA copies/mL, letermovir prophylaxis was stopped and systemic preemptive antiviral treatment was initiated. Peripheral blood for immunoassays was collected at day +30, +60, +90 and +120 after transplantation. The study design is summarized in **Figure 1A**.

For some analyses, we further subdivided alloSCT recipients receiving letermovir prophylaxis or preemptive therapy into non/short-term HCMV reactivating (NSTR) and long-term HCMV reactivating (LTR) patients. NSTR patients were defined as those with no HCMV disease and no more than one HCMV reactivation shorter than three weeks. NSTR patients needed to become PCR negative within three weeks and antiviral treatment needed to be discontinued in the same time period. LTR patients included those with HCMV EOD, more than one reactivation, or a single reactivation exceeding the aforementioned duration. In this study, HCMV reactivation was defined as either two or more consecutive PCR detections with HCMV viral loads falling between 300 and 1000 copies/mL, or a single PCR detection with viral loads equal to or exceeding 1000 copies/mL. HCMV EOD was defined according to the definition of Ljungman and colleagues (15). All patients suffering from HCMV EOD were diagnosed with probable or proven EOD.

### Clinical chart review

The following clinical parameters were recorded: Age, sex, underlying hematological disease, HLA-I matching of the transplant, stem cell source, conditioning regime, administration of antithymocyte globulin, hematopoietic cell transplantation comorbidity index (HCT-CI), HCMV serotype of recipient and donor. Occurrence of acute (a) or chronic (c) graft versus host disease (GvHD), glucocorticosteroid therapy, HCMV load, and mortality were recorded until day +365. Furthermore, complete blood counts (CBC; performed by the institution’s clinical hematology laboratory) were reviewed at the time of blood collection for immunoassays. Clinical characteristics are summarized in **Tables 1**, **S1**, and **S2**.

**Table 1:** Characteristics of HCMV-seropositive patients after allogeneic stem cell transplantation receiving preemptive therapy or letermovir prophylaxis.

Variables	Preemptive Therapy (n = 32)	Letermovir (n = 24)	p*
**Age, median (range)**	55 (23 - 78)	62 (22 - 77)	0.22
Sex, n (%)			
Male	21 (66)	15 (63)	>0.99
Female	11 (34)	9 (37)	
Underlying disease, n (%)			
Chronic leukemia	2 (6)	1 (4)	-
Multiple myeloma	3 (9)	1 (4)	
Acute leukemia	16 (50)	16 (67)	
Lymphoma	2 (6)	1 (4)	
Others	9 (29)	5 (21)	
HLA matching, n (%)			
Matched related	5 (16)	5 (21)	-
Matched unrelated	20 (63)	15 (63)	
Haploidentical	4 (13)	0 (0)	
Mismatch	3 (9)	4 (17)	
Stem cell source, n (%)			
PBSC	32 (100)	24 (100)	1
Conditioning Regimen, n (%)			
Reduced intensity	32 (100)	24 (100)	1
Antithymocyte globulin, n (%)			
No	3 (9)	4 (17)	0.45
Yes	29 (91)	20 (83)	
HCT-CI, n (%)			
0-2	19 (59)	15 (63)	-
3-4	8 (25)	7 (29)	
≥5	5 (16)	2 (8)	
Serostatus (R/D), n (%)			
+/+	27 (84)	18 (75)	0.50
+/-	5 (16)	6 (25)	
aGvHD, n (%)			
0-1	19 (59)	17 (71)	0.41
2-4	13 (41)	7 (29)	
cGvHD, n (%)			
No	27 (84)	20 (83)	>0.99
Yes	5 (16)	4 (17)	
Steroid AUC, median (range)			
by day 100 (mg/kg per day),	0 (0 - 30)	0 (0 - 20)	0.10
One-year mortality, n (%)			
Alive	24 (75)	23 (96)	0.06
Dead	8 (25)	1 (4)	

Mann–Whitney U test or Fisher’s exact test were used as applicable.

aGvHD, acute graft versus host disease; AUC, area under the curve; cGvHD, chronic graft versus host disease; HCT-CI, hematopoietic cell transplantation comorbidity index; HLA, human leukocyte antigen; PBSC, peripheral blood stem cells; R/D, recipient/donor.

### Blood collection, cryopreservation, and thawing process

Monovette blood collection tubes (Sarstedt, Nürnbrecht, Germany) containing ethylenediaminetetraacetic acid (EDTA) were used to collect 36 mL of venous blood. Peripheral blood mononuclear cells (PBMCs) were isolated *via* a density gradient (Histopaque, 1.077 g/mL, Merck, Darmstadt, Germany). After isolation, PBMCs were counted with a Neubauer improved counting chamber (Laboroptik, Lancing, England). Up to 2×10^7^ PBMCs were resuspended in 1.5 mL cryopreservation medium, consisting of 40 % Roswell Park Memorial Institute 1640 Medium (RPMI) Glutamax (Gibco, Thermo Fisher Scientific, Waltham, USA), 50 % fetal calf serum (FCS, Sigma-Aldrich, St. Louis, USA), and 10 % Dimethyl sulfoxide (DMSO, Sigma-Aldrich). PBMCs were initially frozen at -80 °C and transferred to liquid nitrogen for long-term storage.

Cells were thawed in pre-warmed immune cell medium (ICM), consisting of RPMI Glutamax + 10 % FCS + 50 µg/mL Gentamycin (Gibco). Cells were washed with 10 mL of phosphate-buffered saline (PBS, Gibco), resuspended in 10 mL ICM, and rested for 3 h at 37 °C, 5 % CO_2_. Thereafter, the cell suspension was passed through a 70-µm cell strainer (EASYstrainer, Greiner) and adjusted to a concentration of 2.5×10^6^ cells per mL of ICM.

### Preparation and staining of cells for flow cytometry

Flow cytometry was used to analyze global T- and NK-cell phenotypes and to quantify HCMV-specific T cells. Antibodies used for cell staining are summarized in **Supplementary Methods** (**Table S3**). Staining and measurement were performed in 4-mL round-bottom polystyrene tubes (Sarstedt). All centrifugation steps were performed at 300×*g* and 4 °C or room temperature for 5-10 min.

Staining protocol for T-cell phenotyping: 3×10^5^ PBMCs were washed with 1 mL of PBS + 1 % FCS (PBS/F), resuspended in the reflux of the supernatant, and stained for 20 min at RT. Thereafter, cells were washed twice and resuspended in 200 μL PBS/F for analysis.

Staining protocol for NK-cell phenotyping: 5×10^6^ PBMCs were washed with 1 mL of PBS/F and resuspended in 100 μL of blocking buffer, consisting of 80 % WB and 20 % FcR blocking reagent (Miltenyi, Bergisch Gladbach, Germany). After incubation for 10 min at 4 °C, extracellular staining antibodies were added and cells were incubated for another 20 min at 4 °C. Cells were washed twice with PBS/F, resuspended in 250 µL permeabilization solution (10× FACS2 solution [Becton Dickinson, Franklin Lakes, USA], diluted 1:10 in *aqua ad iniectabilia* [Delta Medica, Reutlingen, Germany]), briefly vortexed, and incubated for 12 min at RT. Thereafter, cells were washed twice with PBS/F, resuspended in blocking buffer, and incubated for 10 min at 4 °C. The intracellular staining antibody was added, and cells were incubated for another 20 min at 4 °C. Cells were washed twice with PBS/F and resuspended in 200 μL PBS/F for analysis.

Protocol for HCMV-specific T-cell analysis: PBMCs (5×10^5^ cells in 200 µL ICM) were seeded in a 96-well round bottom plate (Falcon, Corning Incorporated-Life Sciences, Durham, USA) and rested for 2 h (total resting time: 5 h, considering the 3 h resting period after thawing) at 37 °C, 5 % CO_2_. Thereafter, PBMCs were stimulated with either 0.1 µg/mL of a pp65 peptide mix (PepMix HCMVA pp65, >90 % (HPLC-MS) purity, JPT, Berlin, Germany) or 0.1 µg/mL of an HIV (NEF) peptide mix (PepMix HIV-1 NEF, Ultra, JPT, background control). Negative and positive control wells remained unstimulated. After 1 h of incubation at 37 °C, 5 % CO_2_, Brefeldin A (10 µg/mL, Sigma-Aldrich), GolgiStop (1.2 µL per well, Becton Dickinson), and CD107a-APC (2.5 µL per well, Becton Dickinson) were added to all wells. In addition, phorbol myristate acetate (PMA, 0.5 µg/mL) and Ionomycin (1 µg/mL, both Sigma-Aldrich) were added to previously unstimulated wells as a positive control. PBMCs were then incubated for another 16–18 h at 37 °C, 5 % CO_2_. Thereafter, cells were transferred to 4-mL round-bottom polystyrene tubes, washed with PBS/F, and resuspended in PBS/F + 0.5 μg/mL ethidium-monoazide bromide (EMA, Sigma-Aldrich). PBMCs were incubated on ice for 10 min in the dark and for another 10 min illuminated by an LED light. After incubation, cells were washed twice with PBS/F and stained extracellularly for 20 min at 4 °C. After another PBS/F wash step, intracellular staining and analysis of HCMV-specific T cells was performed as described above for NK-cell phenotyping, except for omission of FcR blocking.

### Flow cytometry

Cells were measured using a CytoFLEX cytometer (AS34240) and CytExpert v.2.4 software (both Beckman-Coulter, Brea, USA). Data were analyzed using Kaluza v.2.1 (Beckman Coulter). Gating strategies are shown in **Supplementary Methods** (**Figures S1**, **S2**, **S3**). Frequencies of cell populations determined by flow cytometry were multiplied with the absolute lymphocyte count per μL of whole blood (as determined by clinical CBCs) to estimate the abundance of T-cell and NK-cell subpopulations in peripheral blood. Background-corrected HCMV-specific T-cell frequencies were calculated by subtracting HIV-induced responses (considered unspecific background in HIV-negative individuals) from HCMV-induced responses.

### Statistical analysis

Mann-Whitney *U* test or Fisher’s exact test were used for statistical significance, as indicated in the figure legends. If applicable, Benjamini-Hochberg procedure was used to test for a false-positive discovery rate (FDR) of < 0.2. R was used to perform ROC analysis according to (16). Data were compiled, analyzed, and visualized using Microsoft Excel, GraphPad Prism v.9.2, and R v.4.2.1.

## Results

### Patient characteristics

The cohort for analysis included 56 alloSCT recipients, 32 patients in the preemptive therapy group and 24 patients in the letermovir group. The two groups did not differ significantly in terms of baseline patient characteristics (age, sex, underlying disease, donor type, donor HCMV status, stem cell source, conditioning regimen, HCT-CI score) and duration of follow-up. Detailed patient characteristics are summarized in **Tables 1**, **S1**, and **S2** and the numbers of patients included in each subfigure can be found in **Table S4**.

### HCMV reactivation and viral load

Fourteen out of 32 patients (43.8 %) in the preemptive therapy group showed LTR, while 7 out of the 24 patients (29.2 %) in the letermovir group showed LTR, resulting in comparable reactivation rates until day +365 (p=0.40, **Figure 1B**). Expectedly, letermovir prophylaxis significantly delayed the first HCMV reactivation compared to the preemptive therapy cohort (median: day +171, range: day +30–202 vs. day +33, range: day +5-77, p<0.001, **Figure 1C**). Furthermore, letermovir prophylaxis significantly decreased HCMV peak viral loads until day +120 (median: 0 vs. 1,550 copies/mL, p<0.001) and day +365 (median: 151 vs. 1,550 copies/mL, p=0.043) (**Figure 1D**). Patients receiving preemptive therapy who suffered from LTR had significantly higher HCMV peak loads until day +120 (median: 5,400 vs. 300 copies/mL, p<0.001) and day +365 (median: 5,800 vs. 300 copies/mL, p<0.001) than NSTR patients. Similarly, LTR patients on letermovir prophylaxis showed elevated peak HCMV loads until day +365 compared to NSTR recipients (median: 52,000 vs. 0 copies/mL, p=0.011). There were no differences in HCMV peak loads between NSTR and LTR recipients on letermovir prophylaxis until day +120 (median: 0 vs. 0 copies/mL, p=0.569, **Figure 1E**), indicating successful suppression of viral replication during letermovir intake.

### Reconstitution of the global T- and NK-cell repertoire in alloSCT recipients receiving preemptive therapy or letermovir prophylaxis

**Figure 2:**
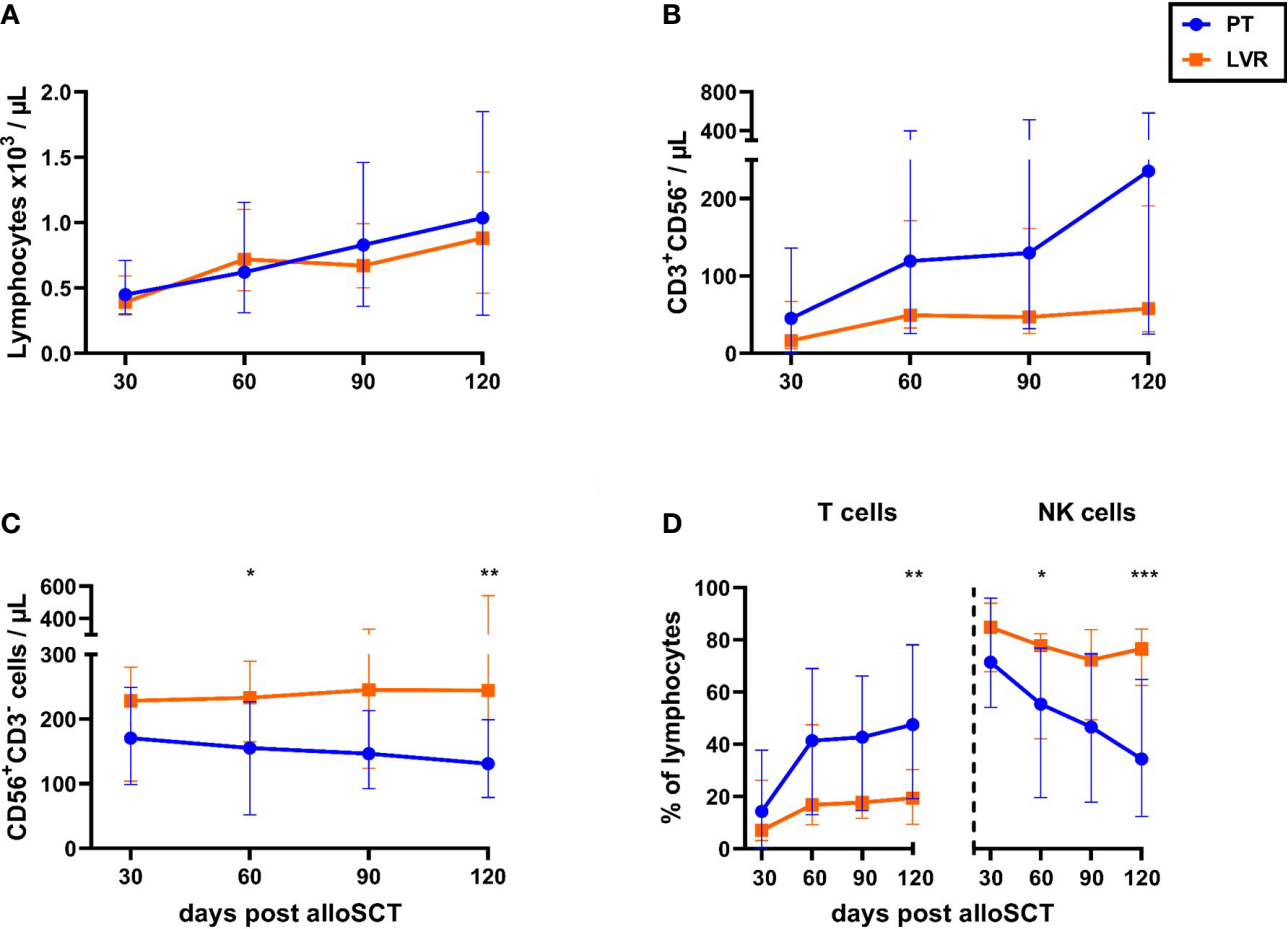
Letermovir prophylaxis enhances NK-cell reconstitution but delays T-cell expansion. **(A-D)** AlloSCT recipients receiving letermovir (LVR, orange squares) or preemptive therapy (PT, blue circles) were compared regarding **(A)** lymphocyte-, **(B)** T-cell- (CD3^+^CD56^-^), and **(C)** NK-cell (CD3^-^CD56^+^) numbers, as well as **(D)** the percentage of T (CD3^+^CD56^-^) and NK cells (CD3^-^CD56^+^) among total lymphocyte numbers. **(A)** Lymphocyte numbers were measured *via* blood count by the routine hematology. **(B-D)** Lymphocyte subpopulations were quantified by flow cytometry. Mann–Whitney *U* test was used to test for statistical significance: * p < 0.05, ** p < 0.01, *** p < 0.001. Median values are shown. Error bars = interquartile ranges. alloSCT, allogeneic stem cell transplantation; CD, cluster of differentiation; LVR, letermovir; PT, preemptive therapy.

In a first step, we compared the immune reconstitution of alloSCT recipients managed with preemptive therapy and letermovir prophylaxis, regardless of the duration of the HCMV reactivation. Antiviral strategies had no impact on absolute lymphocyte counts (**Figure 2A**). However, alloSCT recipients receiving preemptive therapy showed a trend toward higher T-cell counts from day +30 to day +120 than those on letermovir prophylaxis, most markedly at day +120 [median: 235 vs. 58 CD3^+^CD56^-^ T cells/µL, median-to-median ratio (MMR)=4.1, p=0.135, **Figure 2B**]. Inversely, NK-cell counts were higher in patients receiving letermovir prophylaxis at all sampling points, with the largest difference seen at day +120 (median: 244 vs. 131 CD3^-^CD56^+^ NK cells/µL, p=0.006 **Figure 2C**). Together, these findings resulted in an increased T-/NK-cell distribution in preemptive therapy-managed patients compared to patients on letermovir, which was most noticeably at day +120. Preemptive therapy-receiving alloSCT recipients had higher frequencies of circulatory T cells at day +120 (median: 48 % vs. 19 % CD3^+^CD56^-^ T cells of lymphocytes, p=0.003), while letermovir recipients showed higher NK-cell frequencies (median: day +120: 77 % vs. 34 % CD56^+^CD3^-^ NK cells of lymphocytes, p<0.001) (**Figure 2D**).

### T-cell reconstitution in alloSCT recipients managed with preemptive therapy or letermovir prophylaxis

**Figure 3:**
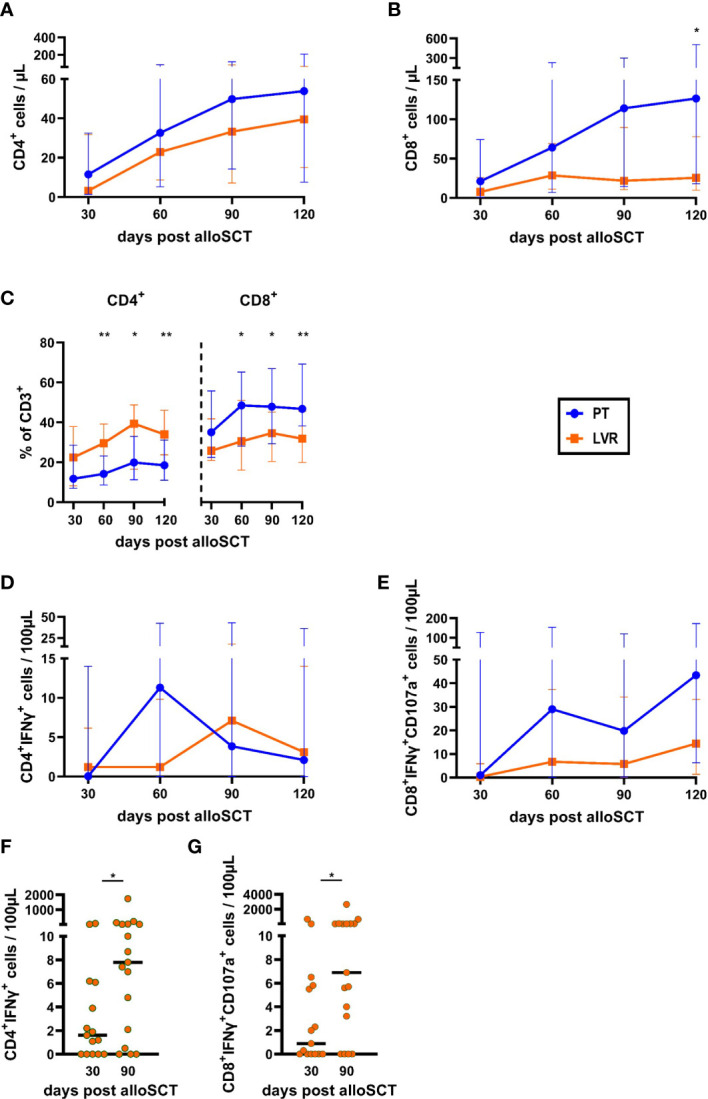
Total and HCMV-specific T-helper cell and cytotoxic T-cell counts, and frequencies are decreased by letermovir prophylaxis. AlloSCT recipients receiving letermovir (LVR, orange squares) or preemptive therapy (PT, blue circles) were compared by flow cytometry. **(A-C)** T-helper (CD4^+^) **(A)** and cytotoxic T-cell (CD8^+^) **(B)** numbers and their percentage of all CD3^+^ cells **(C)** were quantified. **(D, E)** Measurement of background-corrected HCMV-specific **(D)** T-helper cells (CD4^+^IFNγ^+^) and **(E)** cytotoxic T cells (CD8^+^IFNγ^+^ CD107a^+^) after 16-18 h of stimulation with a pp65 HCMV peptide mix. **(F, G) (F)** Numbers of background-corrected HCMV-specific T-helper cells (CD4^+^IFNγ^+^) and **(G)** HCMV-specific cytotoxic T cells (CD8^+^IFNγ^+^ CD107a^+^) at day +30 and +90 in non/short-term HCMV reactivating alloSCT recipients on letermovir prophylaxis. Mann–Whitney *U* test was used to test for statistical significance: * p < 0.05, ** p < 0.01. Median values are shown. Error bars = interquartile ranges. alloSCT, allogeneic stem cell transplantation; CD, cluster of differentiation; LVR, letermovir; PT, preemptive therapy.

CD4^+^ T-helper cell counts were comparable in alloSCT recipients on either antiviral strategy (**Figure 3A**). In contrast, counts of CD8^+^ cytotoxic T lymphocytes were higher in alloSCT recipients managed with preemptive therapy than in the letermovir-treated cohort, reaching significance at day +120 (median: 126 vs. 26 CD8^+^ T cells/µL, p=0.024, **Figure 3B**). As a result, we found a significantly increased CD8^+^/CD4^+^ T-cell ratio in patients receiving preemptive therapy compared to patients on letermovir at day +60, +90, +120 (**Figure 3C**). The distribution of CD4^+^ memory/effector cell subsets (CD4^+^CCR7^+/-^CD45RA^+/-^) was mostly similar between the two treatment groups. However, letermovir recipients had higher naïve CD4^+^ T-cell frequencies than patients managed with preemptive therapy at day +120 (median: 1.8 % vs. 0.8 % CD4^+^CCR7^+^CD45RA^+^ of CD4^+^ T cells, p=0.031, **Figure S4A**).

As expected, quantification of HCMV-specific T cells revealed a trend toward higher numbers of HCMV-specific T cells in alloSCT recipients receiving preemptive therapy compared to patients on letermovir prophylaxis. Compared to patients receiving letermovir, HCMV-specific CD4^+^ T-cell counts were elevated in recipients managed with preemptive therapy at day +60 (median: 11.3 vs. 1.2 CD4^+^IFNγ^+^ T cells/100µL, MMR=9.4, **Figure 3D**), shortly after the median time point of first HCMV reactivation in the preemptive therapy cohort. Additionally, HCMV-specific CD8^+^ T-cell counts were higher in patients receiving preemptive therapy than in those receiving letermovir at all sampling points. This trend was most prominent at day +60 (median: 28.0 vs. 6.7 CD8^+^ IFNγ^+^CD107a^+^ T cells/100µL, MMR=4.2) and day +120 (median: 43.4 vs. 14.4 CD8^+^ IFNγ^+^CD107a^+^ T cells/100µL, MMR=3.0) (**Figure 3E**). Interestingly, even though letermovir recipients showed lower HCMV-specific T-cell numbers than patients managed with preemptive therapy, HCMV-specific T-cell counts expanded from day +30 to day +90 in NSTR letermovir recipients. HCMV-specific CD4^+^ T cells significantly increased from 1.6 CD4^+^IFNγ^+^ T cells/100µL at day +30 to 7.8 CD4^+^IFNγ^+^ T cells/100µL at day +90 (median, p=0.029, **Figure 3F**) in NSTR letermovir patients. Significantly elevated HCMV-specific CD8^+^ T cells from 0.9 CD8^+^ IFNγ^+^CD107a^+^ T cells/100µL at day +30 to 6.9 CD8^+^ IFNγ^+^CD107a^+^ T cells/100µL at day +90 (median, p=0.044, **Figure 3G**) were also found in these patients.

### NK-cell reconstitution in alloSCT recipients receiving preemptive therapy or letermovir prophylaxis

**Figure 4:**
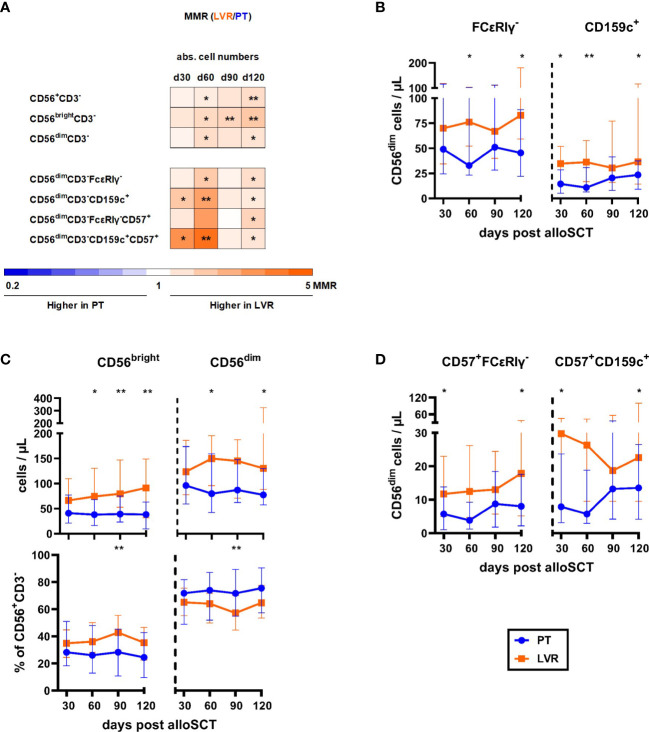
Flow cytometric analysis revealed increased numbers of (“memory-like”) NK-cells in alloSCT patient receiving letermovir prophylaxis compared to preemptive therapy. AlloSCT recipients receiving letermovir (LVR, orange) or preemptive therapy (PT, blue) were compared by flow cytometry. **(A)** Heat map showing the MMR (LVR/PT) of NK-cell and “memory-like” NK-cell counts depending on HCMV management. Orange and blue color intensity indicate higher cell numbers in patients on LVR prophylaxis and patients receiving preemptive therapy, respectively. **(B)** CD56^bright^ (CD56^bright^ CD3^-^), CD56^dim^ (CD56^dim^CD3^-^) and their respective percentages of the total NK-cell (CD56^+^CD3^-^) population. **(C)** Number of “memory-like” CD56^dim^FcϵRIγ^-^ (left) or “memory-like” CD56 ^dim^CD159c^+^ (right) NK-cells. **(D)** Number of mature “memory-like” CD56^dim^FcϵRIγ^-^CD57^+^ (left) or mature “memory-like” CD56^dim^CD159c^+^CD57^+^ (right) NK-cells. Mann-Whitney *U* test and Benjamini-Hochberg procedure to test for a false-positive discovery rate (FDR) of < 0.2 were used to test for statistical significance. * p < 0.05, ** p < 0.01. Median values are shown. Error bars = interquartile ranges. abs., absolute; alloSCT, allogeneic stem cell transplantation; CD, cluster of differentiation; d, day; LVR, letermovir; PT, preemptive therapy; MMR, median-to-median ratio.

Next, we analyzed NK-cell reconstitution. Fold-changes of NK-cell populations in patients receiving letermovir or preemptive therapy are shown in **Figure 4A**. As outlined above, letermovir recipients showed increased numbers of NK cells with the largest differences at day +120 (median: 244 vs. 131 CD3^-^CD56^+^ NK cells/µL, p=0.006, **Figures 2C**, **4A**) compared to preemptive therapy-managed patients. This observation resulted from significantly higher counts of both, CD56^bright^ and CD56^dim^ NK cells (**Figures 4A, B**). We found differences in relative CD56^bright/dim^ proportions between the two cohorts: While letermovir recipients showed higher frequencies of CD56^bright^ NK cells (median: day +90: 43 vs. 28 % CD56^bright^CD3^-^/CD56^+^CD3^-^ NK cells, p=0.009 **Figures 4A, B**), alloSCT recipients receiving preemptive therapy had higher CD56^dim^ NK-cell frequencies (median: day +90: 72 vs. 57 % CD56^dim^CD3^-^/CD56^+^CD3^-^, p=0.009 **Figures 4A, B**). CD56^dim^ NK cells comprise a subset of NK cells that possess “memory-like” characteristics. These NK cells undergo proliferation and display enhanced anti-HCMV properties in response to HCMV infection, thereby contributing to more efficient control of the virus (17–24). Interestingly, “memory-like” (CD56^dim^CD159c^+^(NKG2C)^+^ and CD56^dim^FCϵRIγ^-^) and mature “memory-like” NK-cell (CD56^dim^CD159c^+^ CD57^+^ and CD56^dim^FCϵRIγ^-^CD57^+^) numbers were significantly increased in letermovir recipients compared to patients managed with preemptive therapy (**Figures 4A, C, D**).

### Association of T-cell reconstitution and HCMV control

**Figure 5:**
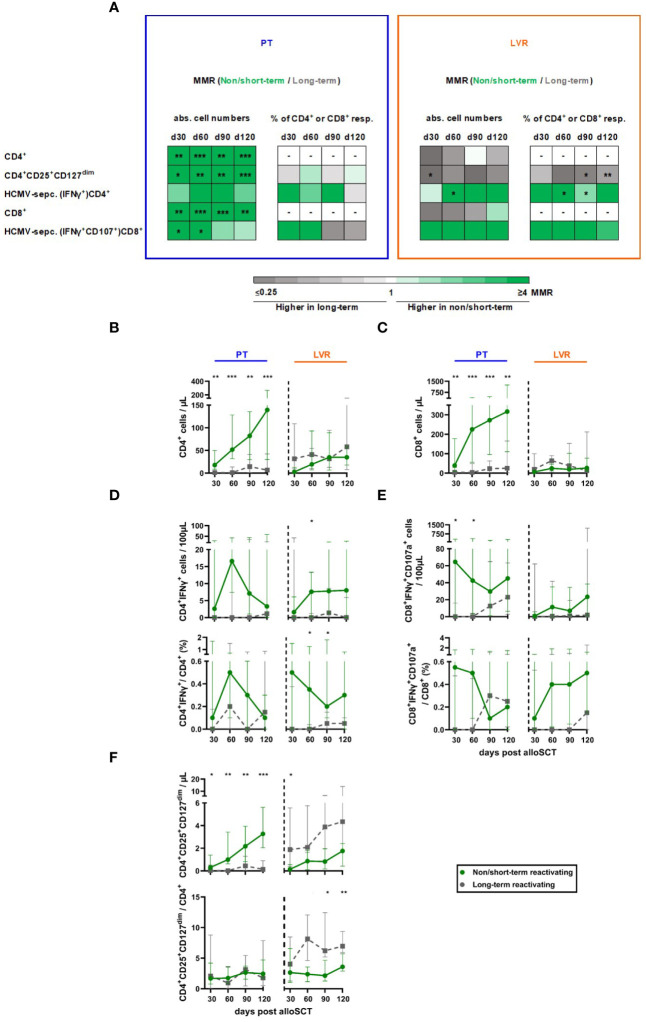
Non/short-term HCMV reactivation in letermovir recipients is associated with an increased HCMV-specific T-helper cell response and decreased total regulatory T cells. Samples from alloSCT recipients, who had non/short-term (green) or long-term (grey) HCMV reactivation and either received letermovir prophylaxis (LVR, orange) or preemptive therapy (PT, blue) were compared by flow cytometry. **(A)** Heat map comparing the MMRs (long-term HCMV reactivating recipients/non/short-term reactivating recipients) of relevant T-cell populations in patients receiving either preemptive therapy (blue square) or letermovir prophylaxis (LVR, orange square) with(out) long-term HCMV reactivation. **(B, C)** Numbers of **(B)** total T-helper cells (CD4^+^) and **(C)** cytotoxic T-cells (CD8^+^). **(D, E)** Background-corrected numbers and percentages of HCMV-specific **(D)** T-helper cells (CD4^+^IFNγ^+^) and **(E)** cytotoxic T-cells (CD8^+^IFNγ^+^ CD107a^+^) after 16-18 h of stimulation with pp65 HCMV peptide mix. **(F)** Numbers and percentages of total regulatory T cells (CD4^+^CD25^+^CD127^dim^). Mann-Whitney *U* test and Benjamini-Hochberg procedure to test for a false-positive discovery rate (FDR) of < 0.2 were used to test for statistical significance. * p < 0.05, ** p < 0.01, *** p < 0.001. Median values are shown. Error bars = interquartile ranges. abs., absolute; alloSCT, allogeneic stem cell transplantation; CD, cluster of differentiation; d, day; LVR, letermovir; MMR, median-to-median ratio; PT, preemptive therapy; resp., respectively.

Next, we compared the lymphocyte reconstitution of alloSCT recipients with NSTR and LTR (**Figure 5A**). NSTR alloSCT recipients managed with preemptive therapy showed significantly higher global CD4^+^ and CD8^+^ T-cell counts compared to LTR recipients, with the highest differences found at day +120 (median: 139.5vs. 6.7 CD4^+^ T cells/µL, p<0.001, **Figures 5A, B**; 316.6 vs. 25.0 CD8^+^ T cells/µL, p=0.002, **Figures 5A, C**). In contrast, global CD4^+^ and CD8^+^ T-cell counts were comparable in patients receiving letermovir prophylaxis with NSTR and LTR (**Figures 5A–C**).

NSTR alloSCT recipients in both cohorts (letermovir and preemptive therapy) had higher HCMV-specific CD4^+^ and CD8^+^ T-cell numbers and frequencies than LTR patients (**Figures 5A, D, E**). Significantly elevated counts of HCMV-specific CD4^+^ were found in NSTR versus LTR patients on letermovir prophylaxis as early as day +60 (median: 7.6 vs. 0 CD4^+^IFNγ^+^ T cells/100µL, p=0.021). Additionally, NSTR letermovir patients showed significantly higher HCMV-specific CD4^+^ frequencies at day +60 (median: 0.35 vs. 0.00 % CD4^+^IFNγ^+^/CD4^+^ T cells, p=0.018) and day +90 (median: 0.20 vs. 0.05 % CD4^+^IFNγ^+^/CD4^+^ T cells, p=0.040) compared to LTR letermovir patients. Moreover, NSTR alloSCT recipients on letermovir prophylaxis showed higher HCMV-specific CD8^+^ T-cell counts and frequencies than LTR patients.

In alloSCT recipients receiving preemptive therapy, regulatory T cells (Tregs, CD4^+^CD25^+^CD127^dim^) expanded in NSTR in contrast to LTR patients. However, relative frequencies of Tregs among CD4^+^ T cells remained comparable between the two groups. In contrast, LTR recipients on letermovir prophylaxis showed increased Treg counts compared to NSTR patients at day +30 (median: 1.9 vs. 0.1 CD4^+^CD25^+^CD127^dim^ T cells/µL, p=0.047). Furthermore, Treg frequencies were significantly elevated in LTR letermovir-treated patients at day +90 (median: 6.2 vs. 2.2 CD4^+^CD25^+^CD127^dim^/CD4^+^ T cells, p=0.019) and day +120 (median: 7.0 vs. 3.6 CD4^+^CD25^+^CD127^dim^ T cells/µL, p=0.003) compared to NSTR patients (**Figure 5F**).

### Association of NK-cell reconstitution and HCMV control

**Figure 6:**
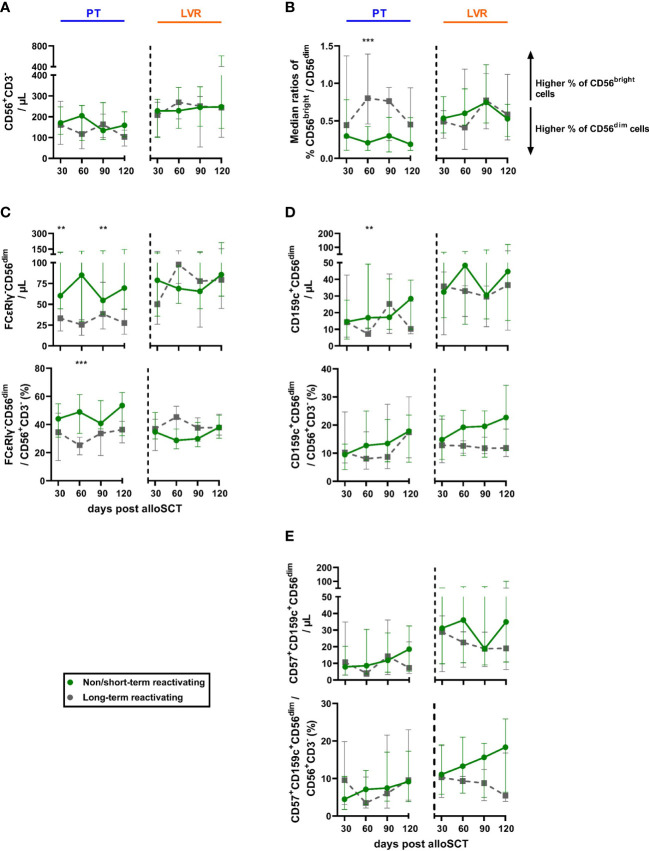
Reconstitution of total NK-cells and “memory-like” NK-cells in non/short-term or long-term HCMV reactivating alloSCT recipients receiving preemptive therapy or letermovir. AlloSCT recipients, who suffered from non/short-term (green) or long-term (grey) HCMV reactivation and either received letermovir prophylaxis (LVR, orange) or preemptive therapy (PT, blue) were compared by flow cytometry. **(A)** NK-cell numbers (CD56^+^CD3^-^). **(B)** Median ratios of CD56^bright^ and CD56^dim^ NK-cells. **(C-E)** Absolute cell numbers and percentages of **(C)** FcϵRIγ^-^CD56^dim^, **(D)** CD159c^+^CD56^dim^, and **(E)** CD57^+^CD159c^+^CD56^dim^ NK cells. Mann–Whitney *U* test was used to test for statistical significance: * p < 0.05, ** p < 0.01. Median values are shown. Error bars = interquartile ranges. alloSCT, allogeneic stem cell transplantation; CD, cluster of differentiation; d, day; LVR, letermovir, PT, preemptive therapy.

NK-cell numbers were similar between NSTR and LTR recipients with both antiviral strategies (**Figure 6A**). However, while preemptive therapy-managed alloSCT recipients with LTR showed elevated CD56^bright^/CD56^dim^ NK-cell ratios compared to NSTR patients (median: day +60: 0.80 vs. 0.21, p<0.001), this trend was not seen in letermovir recipients (**Figure 6B**).

“Memory-like” (CD56^dim^CD159c(NKG2C)^+^ and CD56^dim^FCϵRIγ^-^) NK-cell numbers and CD56^dim^FCϵRIγ^-^ frequencies were elevated in NSTR alloSCT recipients receiving preemptive therapy compared to LTR patients (**Figures 6C, D**). In contrast, we did not find statistically significant differences in “memory-like” or mature “memory-like” (CD56^dim^CD159c^+^CD57^+^) NK-cell numbers between patients on letermovir prophylaxis with and without LTR (**Figures 6C–E**). However, NSTR patients on letermovir prophylaxis showed strong trends toward higher CD56^dim^CD159c^+^ (median: 22.7 vs. 11.8 % CD56^dim^CD159c^+^/CD56^+^CD3^-^, p=0.089) and CD56^dim^CD159c^+^CD57^+^ (median: 18.3 vs. 5.4 % CD56^dim^CD159c^+^CD57^+^/CD56^+^CD3^-^, p=0.065) frequencies compared to LTR recipients at day +120 (**Figures 6D, E**).

### Identification of immune markers predicting HCMV control and reactivations

**Figure 7:**
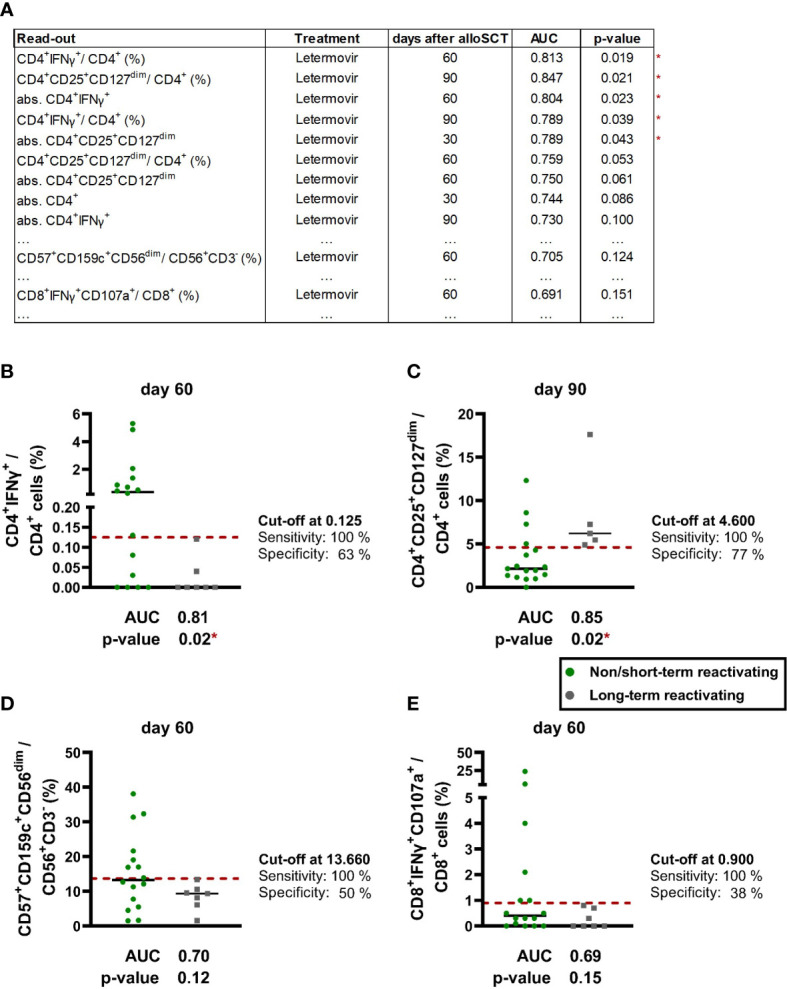
ROC-analysis revealed CD4^+^ T cells as promising biomarker for future long-term HCMV reactivations. **(A)** ROC analysis was performed for numbers and frequencies of the following populations of alloSCT recipients with non/short-term or long-term HCMV reactivation(s) on letermovir prophylaxis: Global T cells (CD3^+^, CD4^+^, CD8^+^, CD4^+^CD25^+^CD127^+^), HCMV-specific T cells (CD4^+^IFNγ^+^, CD8^+^IFNγ^+^CD107a^+^), global NK cells (CD56^+^CD3^-^) and “memory-like” NK cells [(CD57^+^)CD159c^+^CD56^dim^ and (CD57^+^) FcϵRIγ^-^CD56dim]. The table shows results with a p-value ≤ 0.1 as well as the lowest p-value for “memory-like” NK cells and HCMV-specific CD8^+^ T cells. **(B-D)** ROC analysis of selected cell frequencies of non/short-term (green dots) and long-term (grey dots) HCMV reactivating patients on letermovir prophylaxis: **(B)** HCMV-specific CD4^+^ T cells, **(C)** regulatory T cells, **(D)** “memory-like” NK cells, **(E)** HCMV-specific CD8^+^ T cells. Results with a significant Mann-Whitney *U* test and non-significant Benjamini-Hochberg procedure to test for a false-positive discovery rate (FDR) of < 0.2 were marked as * p < 0.05. Black lines = median. alloSCT, allogeneic stem cell transplantation; CD, cluster of differentiation; IFN, Interferon; ROC, receiver operating characteristic.

Finally, we performed ROC analysis to identify the most promising biomarkers on days +30, +60, and +90 to predict future LTR during letermovir prophylaxis (**Figure 7A**). The strongest predictor of LTR were low HCMV-specific CD4^+^ T cells and high regulatory CD4^+^ T cells at day +60 and day +90 (**Figure 7A**). Specifically, frequencies of HCMV-specific CD4^+^ T cells at day +60 were the best discriminator between NSTR and LTR letermovir-treated recipients [median: 0.35 vs. 0.00 % CD4^+^IFNγ^+^/CD4^+^, p=0.019, area under the curve (AUC)=0.81, **Figure 7B**], closely followed by Tregs at day +90 (median: 2.2 vs. 6.2 % CD4^+^CD25^+^CD127^dim^/CD4^+^. p=0.021, AUC=0.85, **Figure 7C**). In addition, frequencies of CD57^+^CD159c^+^CD56^dim^ memory-like NK cells at day +60 yielded relatively good yet non-significant discrimination between LTR (9.4 %) and NSTR (13.3 %) letermovir recipients (p=0.124, **Figure 7D**). In contrast, HCMV-specific CD8^+^ reconstitution was less predictive for HCMV control (**Figure 7E**).

## Discussion

A better understanding of the influence of letermovir on immune reconstitution and the identification of immune markers to predict HCMV control will be essential to improve and individualize HCMV prophylaxis and management in alloSCT recipients. Letermovir prophylaxis decreases the HCMV load and delays the first HCMV reactivation (8, 25). Therefore, the immune system is given additional time to reconstitute an efficient immune response to prevent LTR. However, the lack of HCMV reactivation during letermovir prophylaxis and lack of immune exposure to HCMV antigens can alter the reconstitution of T cells and NK cells.

Compared to patients receiving preemptive therapy, we found that letermovir prophylaxis decreased T-cell numbers but led to higher NK-cell numbers. In line with the literature, we found increased numbers of “memory-like” NK cells in NSTR compared to LTR patients receiving preemptive therapy (18, 23, 24). Interestingly, we also found a strong trend towards increased frequencies of CD159c^+^ and mature CD57^+^CD159c^+^ “memory-like” NK cells in NSTR vs. LTR patients in the letermovir group. Thus, CD159c^+^ NK cells and their strong potential for expansion, cytokine secretion, and cytotoxicity might help to control the virus after discontinuation of letermovir (17–20). It is likely that HCMV reactivation events, which would be expected to occur predominantly after the end of our observation period, will further shape the “memory-like” NK-cell repertoire. Future research should determine how “memory-like” NK cells can expand despite letermovir and clarify their role in late-onset HCMV disease after discontinuation of letermovir prophylaxis.

As many other viral infections, HCMV reactivations result in clonal expansion of CD4^+^ and CD8^+^ T cells (26–28). Consistent with recent publications, we found that letermovir prophylaxis decreases the expansion of T cells compared to preemptive therapy. This observation likely results from decreased proliferation of HCMV-specific CD8^+^- and CD4^+^ T cells due to impaired HCMV antigen exposure resulting from inhibition of HCMV replication by letermovir (8, 9, 29). Consequently, letermovir results in an altered CD8^+^/CD4^+^ ratio. CD4^+^ T cells are more abundant than CD8^+^ T cells in most HCMV-seronegative healthy adults, whereas HCMV infection commonly inverts this ratio (30, 31). Therefore, our results align with the expectation of higher CD8^+^/CD4^+^ ratios in the early post-transplant stage in patients managed with preemptive therapy than in those receiving letermovir. However, we hypothesize that delayed expansion of CD8^+^ T cells may occur after discontinuation of letermovir prophylaxis and subsequent (sub)clinical HCMV reactivation. Reactivation of HCMV has been linked to post-transplant immune dysfunction, including defects in the T-cell compartment (32–35). It is interesting to speculate that inhibition of HCMV replication by letermovir prophylaxis might prevent these negative effects on the T-cell compartment. The median 71-day delay in HCMV reactivation following letermovir discontinuation observed in our patient cohort could be attributed to this effect, and it may also contribute to reduced transplant-related mortality associated with letermovir prophylaxis (12, 13, 32). In addition, HCMV-specific T cells proliferated in NSTR patients during letermovir prophylaxis. Potential explanations for this observation could be the presentation of HCMV antigens that are expressed early in the HCMV replication cycle, cross reactivity, or minor, subclinical HCMV reactivations despite letermovir. In contrast, patients with LTR did not show a significant increase in HCMV-specific T-cell numbers during letermovir administration.

Notably, our finding that NSTR alloSCT recipients receiving letermovir had significantly higher frequencies of HCMV-specific CD4^+^ T cells than LTR patients indicate that antigen-specific CD4^+^ T-cell responses are pivotal to suppress post-transplant HCMV reactivations. This finding aligns with numerous publications that showed a protective effect of CD4^+^ T cells in the resolution of primary HCMV infection, prevention of prolonged HCMV shredding, and development of a functional memory response against HCMV (26, 36–38). In alloSCT recipients, an early and robust global and HCMV-specific CD4^+^ T-cell reconstitution is associated with reduced HCMV viremia and HCMV disease (39–41). Consequently, adoptive HCMV-specific T-cell transfer has been proposed to fight recurrent, treatment-resistant HCMV reactivation. Of note, prior studies suggested that CD4^+^ T-cell reconstitution has to precede and subsequently orchestrate CD8^+^ T-cell reconstitution in order to establish increased protection against HCMV diseases (42–46). Due to the inhibition of HCMV replication by letermovir, CD4^+^ T cells have more time to reconstitute before the first HCMV reactivation and might be a major contributor to the less frequent LTR in letermovir patients (8, 25).

Even though the protective effect of the CD4^+^ T cells against HCMV is apparent, our results imply that closer characterization of the HCMV-specific CD4^+^ T-cell response may help to identify patients at risk for late-onset HCMV disease. The anti-HCMV CD4^+^ T-cell repertoire consists of three main populations: CD4^+^ cytolytic T cells, CD4^+^ memory T cells and Tregs (26, 47). Patients receiving preemptive therapy, who only showed NSTR, had increased numbers of all three populations, indicating a balanced expansion of T-cell populations compared to the very limited T-cell expansion in LTR patients. In contrast, we found significantly increased numbers and frequencies of total Tregs in LTR patients on letermovir prophylaxis compared to NSTR patients, suggesting that Tregs might promote long-term HCMV reactivation in a setting of letermovir prophylaxis. Tregs are the most common HCMV-specific CD4^+^ subtype in HCMV-seropositive healthy individuals (47). They modulate the response of conventional T cells in order to establish self-tolerance and prevent tissue damage (48, 49) but also inhibit the establishment of conventional T-cell responses in patients with HCMV and other viral infections (48, 50–52). For instance, depletion of Tregs from PBMCs resulted in increased IFNγ production of T cells in response to HCMV antigens (50). Similarly, depletion of Tregs after establishment of murine (M)CMV latency enhanced MCMV-specific CD4^+^- and CD8^+^ T-cell numbers and counteracted splenic MCMV latency in a mouse model (50). Given these immunomodulatory Treg functions, alterations in the patients’ total Treg repertoire could also explain recent reports of less common cGvHD in patients receiving letermovir prophylaxis (29, 53).

The differentiation and expansion of Tregs is known to be facilitated by antigen stimulation in combination with lacking innate immune response and co-stimulation (54). In patients on letermovir prophylaxis, HCMV replication below the PCR threshold and/or the presentation of early HCMV antigens could lead to low-level HCMV antigen presentation. In these scenarios, limited inflammation could result in a lack of secondary and tertiary T-cell stimulation signals. This might create an immune environment favoring HCMV-specific Treg differentiation and expansion. These Tregs could in turn inhibit the conventional T-cell response needed for HCMV control, potentially contributing to HCMV reactivation after discontinuation of letermovir. However, we only measured total Tregs and it remains to be determined whether the total Treg population correlates with HCMV-specific Tregs.

Our finding of high CD4^+^ T-cell counts and low Treg frequencies being major predictors of HCMV control after letermovir prophylaxis could have translational significance. Assays designed to quantify the IFNγ release after stimulation of PBMCs or whole blood with various antigens [so-called Interferon-γ release assays (IGRAs)] are commercially available and have proven their ability to predict the risk of long-term HCMV reactivations in alloSCT recipients during preemptive therapy (55–58). Our data imply that IGRA measurements might also be beneficial for risk stratification before discontinuation of letermovir prophylaxis. Given the stronger association of HCMV-specific CD4^+^ cells with NSTR compared to HCMV-specific CD8^+^ cells in our study, it would be useful to develop or optimize distinct stimulation pools for both major T-cell populations. An immunoassay kit with two separate stimulation pools for CD4^+^ and CD8^+^ T cells is already available for tuberculosis and HCMV (59, 60), underscoring the feasibility of such an approach. In addition, efforts have been made to diversify cytokine readouts for other infective diseases (61–63). In view of our present data, addition of specific Treg cytokine readouts such as TGFβ and IL10 might be particularly beneficial. Such refined assays could contribute to the identification of patients with a high risk for HCMV reactivation and disease and thereby facilitate risk-adapted clinical management approaches, including closer follow-up monitoring and individualized duration of letermovir prophylaxis. Extended letermovir prophylaxis from 100 to 200 days has demonstrated to decrease clinical significant HCMV events in a double-blinded placebo-controlled clinical trial (ClinicalTrials.gov, Identifier: NCT03930615) (64). However, limitation of extended prophylaxis to alloSCT recipients at particularly high risk for LTR and disease, identified by immunoassays, might be an attractive and possibly more cost-effective future approach.

Our study has some limitations. Firstly, the observation period of the study was limited to 120 days post-transplant. Thus, effects of HCMV reactivation after discontinuation of letermovir were not captured. In addition, only seven patients developed LTR after letermovir prophylaxis. Groups were well-matched for relevant patient characteristics; however, it is conceivable that additional clinical factors (e.g., HCMV serotypes of recipient and donor) may have influenced immune reconstitution and the duration of the HCMV reactivation after alloSCT (4, 65, 66).

There are additional technical limitations. PBMCs were analyzed after cryopreservation. Although common practice, it is known that cryopreservation can alter T-cell phenotypes and antigen-specific T-cell responses (63, 67–69). Moreover, absolute numbers of specific cell subsets were approximated using a combination of flow cytometry and lymphocyte counts measured by routine hematology. Therefore, these cell numbers should be considered an estimate. In addition, HCMV-specific T cells were measured after PBMC stimulation with HCMV antigen. Quantification *via* multimers could have provided a more precise estimate of total HCMV-specific T cells in the peripheral blood. However, unlike multimers, our stimulation approach was not HLA-restricted and specifically quantified functional HCMV-specific T cells. Thus, we argue that our approach is more suited to detect functional anti-HCMV T-cell response.

Despite limitations, we herein showed significant changes in T-cell reconstitution in alloSCT on letermovir prophylaxis when compared to patients managed with preemptive therapy. Furthermore, we provided inaugural evidence that letermovir prophylaxis also alters NK-cell reconstitution. Importantly, we identified HCMV-specific CD4^+^ T cells as a major predictor of NSTR in letermovir recipients. In contrast to patients receiving preemptive therapy, increased Treg numbers and frequencies were associated with LTR. In the future, HCMV-specific CD4^+^ responses in alloSCT patients on letermovir prophylaxis should be studied prospectively in larger patient cohorts to evaluate whether HCMV-specific CD4^+^ T cells and Tregs are predictive biomarkers for the risk of HCMV reactivation following cessation of antiviral prophylaxis. This might allow clinicians to prolong the duration of letermovir prophylaxis for patients at high risk for LTR and to further decrease morbidity and mortality associated with HCMV reactivations and disease. Finally, future studies should include extended observation periods to investigate immune reconstitution and anti-HCMV response in alloSCT recipients after discontinuation of letermovir prophylaxis and potential HCMV reactivation.

## Data availability statement

The raw data supporting the conclusions of this article will be made available by the authors, without undue reservation.

## Ethics statement

The studies involving human participants were reviewed and approved by Ethics Committees of the University of Wuerzburg (protocol code 17/19-sc) (Ethik-Kommission der Medizinischen Fakultät, Bau C12, Josef-Schneider-Str. 4, 97080 Würzburg). The patients/participants provided their written informed consent to participate in this study.

## Author contributions

The study was conceived by HE, GG, and SK. Patient enrollment and clinical documentation were performed by DG and SK. Experiments were planed and performed by CL, AR, and CK. Data were analyzed by CL, IM, FE, and SK. Data were visualized by CL. Project administration and supervision were led by CL, FE, LD, HE, SW, and SK. Funding was acquired by FE, LD, HE, GG, and SK. The original draft was written by CL, SW, and SK. All authors contributed to the article and approved the submitted version.

## Supplementary material

Supplementary Figure 1 Used gating strategy to study the T-cell phenotype. CD, cluster of differentiation, CM, central memory, EM, effector memory, EMRA, effector memory cells re-expressing CD45RA, Treg, regulatory T-cells.

Supplementary Figure 2 Used gating strategy to study the NK-cell phenotype. CD, cluster of differentiation.

Supplementary Figure 3 Used gating strategy to study HCMV-specific T cells after stimulation. CD, cluster of differentiation.

Supplementary Figure 4 Memory T-cell distribution in alloSCT recipients receiving letermovir prophylaxis or preemptive therapy. **(A)** Distribution of T-helper cells (CD4^+^): T_naïve_ (CCR7^+^CD45RA^+^), T_cm_ (CCR7^+^CD45RA^-^), T_em_ (CCR7^-^CD45RA^-^), T_EMRA_ (CCR7^-^CD45RA^+^). alloSCT, allogeneic stem cell transplantation, CD, cluster of differentiation, LVR, letermovir, PT, preemptive therapy, T_cm_, central memory T cell, T_em_, effector memory T cell, T_EMRA_, effector memory cells re-expressing CD45RA, T_naïve_, naïve T cells.

Supplementary Table 1 Patient characteristics of non/short-term and long-term HCMV reactivating patients after allogeneic stem cell transplantation receiving preemptive therapy.

Supplementary Table 2 Patient characteristics of non/short-term and long-term HCMV reactivating patients after allogeneic stem cell transplantation receiving letermovir prophylaxis.

Supplementary Table 3 Antibodies used for flow cytometry.

Supplementary Table 4 Patient numbers per (sub)figure.

## References

[1] Singh AK McGuirk JP. Allogeneic stem cell transplantation: a historical and scientific overview. Cancer Res (2016) 76(22):6445–51. doi: 10.1158/0008-5472.CAN-16-1311 27784742

[2] Broers AE van der Holt R van Esser JW Gratama JW Henzen-Logmans S Kuenen-Boumeester V. Increased transplant-related morbidity and mortality in CMV-seropositive patients despite highly effective prevention of CMV disease after allogeneic T-cell-depleted stem cell transplantation. Blood (2000) 95(7):2240–5. doi: 10.1182/blood.V95.7.2240 10733491

[3] Latgé JP Chamilos G. Aspergillus fumigatus and aspergillosis in 2019. Clin Microbiol Rev (2019) 33(1):e00140–18. doi: 10.1128/CMR.00140-18 PMC6860006 31722890

[4] Stern L Withers B Avdic S Gottlieb D Abendroth A Blyth E. Human cytomegalovirus latency and reactivation in allogeneic hematopoietic stem cell transplant recipients. Front Microbiol (2019) 10:1186. doi: 10.3389/fmicb.2019.01186 31191499 PMC6546901

[5] Griffiths P Reeves M. Pathogenesis of human cytomegalovirus in the immunocompromised host. Nat Rev Microbiol (2021) 19(12):759–73. doi: 10.1038/s41579-021-00582-z PMC8223196 34168328

[6] Brillantes M Beaulieu AM. Memory and memory-like NK cell responses to microbial pathogens. Front Cell Infect Microbiol (2020) 10:102. doi: 10.3389/fcimb.2020.00102 32269968 PMC7109401

[7] Capuano C Pighi C Battella S De Federicis D Galandrini R Palmieri G. Harnessing CD16-mediated NK cell functions to enhance therapeutic efficacy of tumor-targeting mAbs. Cancers (Basel) (2021) 13(10):2500. doi: 10.3390/cancers13102500 34065399 PMC8161310

[8] Zamora D Duke ER Xie H Edmison BC Akoto B Kiener R. Cytomegalovirus-specific T-cell reconstitution following letermovir prophylaxis after hematopoietic cell transplantation. Blood (2021) 138(1):34–43. doi: 10.1182/blood.2020009396 33657225 PMC8493975

[9] Sperotto A Candoni A Gottardi M Facchin G Stella R De Marchi R. Cytomegalovirus prophylaxis versus pre-emptive strategy: different CD4(+) and CD8(+) T cell reconstitution after allogeneic hematopoietic stem cell transplantation. Transplant Cell Ther (2021) 27(6):518.e1–.e4. doi: 10.1016/j.jtct.2021.03.003 33812803

[10] Einsele H Ehninger G Hebart H Wittkowski KM Schuler U Jahn G. Polymerase chain reaction monitoring reduces the incidence of cytomegalovirus disease and the duration and side effects of antiviral therapy after bone marrow transplantation. Blood (1995) 86(7):2815–20. doi: 10.1182/blood.V86.7.2815.2815 7670117

[11] Ljungman P Loré K Aschan J Klaesson S Lewensohn-Fuchs I Lönnqvist B. Use of a semi-quantitative PCR for cytomegalovirus DNA as a basis for pre-emptive antiviral therapy in allogeneic bone marrow transplant patients. Bone Marrow Transplant (1996) 17(4):583–7. 8722359

[12] Chemaly RF Ullmann AJ Stoelben S Richard MP Bornhäuser M Groth C. Letermovir for cytomegalovirus prophylaxis in hematopoietic-cell transplantation. N Engl J Med (2014) 370(19):1781–9. doi: 10.1056/NEJMoa1309533 24806159

[13] Marty FM Ljungman P Chemaly RF Maertens J Dadwal SS Duarte RF. Letermovir prophylaxis for cytomegalovirus in hematopoietic-cell transplantation. N Engl J Med (2017) 377(25):2433–44. doi: 10.1056/NEJMoa1706640 29211658

[14] Cassaniti I Colombo AA Bernasconi P Malagola M Russo D Iori AP. Positive HCMV DNAemia in stem cell recipients undergoing letermovir prophylaxis is expression of abortive infection. Am J Transplant (2021) 21(4):1622–8. doi: 10.1111/ajt.16450 33320429

[15] Ljungman P Boeckh M Hirsch HH Josephson F Lundgren J Nichols G. Definitions of cytomegalovirus infection and disease in transplant patients for use in clinical trials. Clin Infect Diseases (2017) 64(1):87–91. doi: 10.1093/cid/ciw668 27682069

[16] Hanley JA McNeil BJ. The meaning and use of the area under a receiver operating characteristic (ROC) curve. Radiology (1982) 143(1):29–36. doi: 10.1148/radiology.143.1.7063747 7063747

[17] Barron MA Gao D Springer KL Patterson JA Brunvand MW McSweeney PA. Relationship of reconstituted adaptive and innate cytomegalovirus (CMV)-specific immune responses with CMV viremia in hematopoietic stem cell transplant recipients. Clin Infect Dis (2009) 49(12):1777–83. doi: 10.1086/648423 19911937

[18] Basílio-Queirós D Venturini L Luther-Wolf S Dammann E Ganser A Stadler M. Adaptive NK cells undergo a dynamic modulation in response to human cytomegalovirus and recruit T cells in *in vitro* migration assays. Bone Marrow Transplantation (2022) 57(5):712–20. doi: 10.1038/s41409-022-01603-y PMC9090630 35177828

[19] Foley B Cooley S Verneris MR Pitt M Curtsinger J Luo X. Cytomegalovirus reactivation after allogeneic transplantation promotes a lasting increase in educated NKG2C+ natural killer cells with potent function. Blood (2012) 119(11):2665–74. doi: 10.1182/blood-2011-10-386995 PMC3311280 22180440

[20] Foley B Cooley S Verneris MR Curtsinger J Luo X Waller EK. Human cytomegalovirus (CMV)-induced memory-like NKG2C(+) NK cells are transplantable and expand *in vivo* in response to recipient CMV antigen. J Immunol (2012) 189(10):5082–8. doi: 10.4049/jimmunol.1201964 PMC3490031 23077239

[21] Zhang T Scott JM Hwang I Kim S. Cutting edge: antibody-dependent memory-like NK cells distinguished by FcRγ deficiency. J Immunol (2013) 190(4):1402–6. doi: 10.4049/jimmunol.1203034 PMC3623944 23345329

[22] Hwang I Zhang T Scott JM Kim AR Lee T Kakarla T. Identification of human NK cells that are deficient for signaling adaptor FcRγ and specialized for antibody-dependent immune functions. Int Immunol (2012) 24(12):793–802. doi: 10.1093/intimm/dxs080 22962434 PMC3621379

[23] Cichocki F Taras E Chiuppesi F Wagner JE Blazar BR Brunstein C. Adaptive NK cell reconstitution is associated with better clinical outcomes. JCI Insight (2019) 4(2). doi: 10.1172/jci.insight.125553 PMC6413795 30674718

[24] Park KH Ryu JH Bae H Yun S Jang JH Han K. Delayed NK cell reconstitution and reduced NK activity increased the risks of CMV disease in allogeneic-hematopoietic stem cell transplantation. Int J Mol Sci (2020) 21(10):3663. doi: 10.3390/ijms21103663 32455959 PMC7279475

[25] Mori Y Jinnouchi F Takenaka K Aoki T Kuriyama T Kadowaki M. Efficacy of prophylactic letermovir for cytomegalovirus reactivation in hematopoietic cell transplantation: a multicenter real-world data. Bone Marrow Transplantation (2021) 56(4):853–62. doi: 10.1038/s41409-020-01082-z 33139867

[26] Lim EY Jackson SE Wills MR. The CD4+ T cell response to human cytomegalovirus in healthy and immunocompromised people. Front Cell Infect Mi (2020) 10:202. doi: 10.3389/fcimb.2020.00202 PMC7248300 32509591

[27] Degli-Esposti MA Hill GR. Immune control of cytomegalovirus reactivation in stem cell transplantation. Blood (2022) 139(9):1277–88. doi: 10.1182/blood.2020010028 34166512

[28] Jagadeesh A Prathyusha AMVN Sheela GM Bramhachari PV. T Cells in viral infections: the myriad flavours of antiviral immunity. In: Bramhachari PV, editor. Dynamics of immune activation in viral diseases. Singapore: Springer Singapore (2020). p. 139–48.

[29] Gabanti E Borsani O Colombo AA Zavaglio F Binaschi L Caldera D. Human cytomegalovirus-specific T-cell reconstitution and late-onset cytomegalovirus infection in hematopoietic stem cell transplantation recipients following letermovir prophylaxis. Transplant Cell Ther (2022) 28(4):211.e1–.e9. doi: 10.1016/j.jtct.2022.01.008 35042012

[30] Chidrawar S Khan N Wei W McLarnon A Smith N Nayak L. Cytomegalovirus-seropositivity has a profound influence on the magnitude of major lymphoid subsets within healthy individuals. Clin Exp Immunol (2009) 155(3):423–32. doi: 10.1111/j.1365-2249.2008.03785.x PMC2669518 19220832

[31] Wertheimer AM Bennett MS Park B Uhrlaub JL Martinez C Pulko V. Aging and cytomegalovirus infection differentially and jointly affect distinct circulating T cell subsets in humans. J Immunol (2014) 192(5):2143–55. doi: 10.4049/jimmunol.1301721 PMC3989163 24501199

[32] Suessmuth Y Mukherjee R Watkins B Koura DT Finstermeier K Desmarais C. CMV reactivation drives posttransplant T-cell reconstitution and results in defects in the underlying TCRβ repertoire. Blood (2015) 125(25):3835–50. doi: 10.1182/blood-2015-03-631853 PMC4473113 25852054

[33] Itzykson R Robin M Moins-Teisserenc H Delord M Busson M Xhaard A. Cytomegalovirus shapes long-term immune reconstitution after allogeneic stem cell transplantation. Haematologica (2015) 100(1):114–23. doi: 10.3324/haematol.2014.113415 PMC4281324 25261095

[34] Lugthart G van Ostaijen-Ten Dam MM Jol-van der Zijde CM van Holten TC Kester MG Heemskerk MH. Early cytomegalovirus reactivation leaves a specific and dynamic imprint on the reconstituting T cell compartment long-term after hematopoietic stem cell transplantation. Biol Blood Marrow Transplant (2014) 20(5):655–61. doi: 10.1016/j.bbmt.2014.01.018 24462981

[35] Politikos I Lavery JA Hilden P Cho C Borrill T Maloy MA. Robust CD4+ T-cell recovery in adults transplanted with cord blood and no antithymocyte globulin. Blood Adv (2020) 4(1):191–202. doi: 10.1182/bloodadvances.2019000836 31935291 PMC6960461

[36] Einsele H Roosnek E Rufer N Sinzger C Riegler S Löffler J. Infusion of cytomegalovirus (CMV)-specific T cells for the treatment of CMV infection not responding to antiviral chemotherapy. Blood (2002) 99(11):3916–22. doi: 10.1182/blood.V99.11.3916 12010789

[37] Sester M Sester U Gartner B Heine G Girndt M Mueller-Lantzsch N. Levels of virus-specific CD4 T cells correlate with cytomegalovirus control and predict virus-induced disease after renal transplantation. Transplantation (2001) 71(9):1287–94. doi: 10.1097/00007890-200105150-00018 11397964

[38] Antoine P Olislagers V Huygens A Lecomte S Liesnard C Donner C. Functional exhaustion of CD4+ T lymphocytes during primary cytomegalovirus infection. J Immunol (2012) 189(5):2665–72. doi: 10.4049/jimmunol.1101165 22865914

[39] Einsele H Ehninger G Steidle M Fischer I Bihler S Gerneth F. Lymphocytopenia as an unfavorable prognostic factor in patients with cytomegalovirus infection after bone marrow transplantation. Blood (1993) 82(5):1672–8. doi: 10.1182/blood.V82.5.1672.bloodjournal8251672 8395913

[40] Avetisyan G Larsson K Aschan J Nilsson C Hassan M Ljungman P. Impact on the cytomegalovirus (CMV) viral load by CMV-specific T-cell immunity in recipients of allogeneic stem cell transplantation. Bone Marrow Transplant (2006) 38(10):687–92. doi: 10.1038/sj.bmt.1705507 17001346

[41] Lilleri D Gerna G Fornara C Lozza L Maccario R Locatelli F. Prospective simultaneous quantification of human cytomegalovirus-specific CD4+ and CD8+ T-cell reconstitution in young recipients of allogeneic hematopoietic stem cell transplants. Blood (2006) 108(4):1406–12. doi: 10.1182/blood-2005-11-012864 16614242

[42] Walter EA Greenberg PD Gilbert MJ Finch RJ Watanabe KS Thomas ED. Reconstitution of cellular immunity against cytomegalovirus in recipients of allogeneic bone marrow by transfer of T-cell clones from the donor. N Engl J Med (1995) 333(16):1038–44. doi: 10.1056/NEJM199510193331603 7675046

[43] Rentenaar RJ Gamadia LE van der Hoek N van Diepen FN Boom R Weel JF. CD4(+) T-cell dynamics in primary cytomegalovirus infection. Transplant Proc (2001) 33(3):2313–4. doi: 10.1016/S0041-1345(01)02004-8 11377542

[44] Rentenaar RJ Gamadia LE van DerHoek N van Diepen FN Boom R Weel JF. Development of virus-specific CD4(+) T cells during primary cytomegalovirus infection. J Clin Invest (2000) 105(4):541–8. doi: 10.1172/JCI8229 PMC289159 10683384

[45] Gamadia LE Remmerswaal EB Weel JF Bemelman F van Lier RA Ten Berge IJ. Primary immune responses to human CMV: a critical role for IFN-gamma-producing CD4+ T cells in protection against CMV disease. Blood (2003) 101(7):2686–92. doi: 10.1182/blood-2002-08-2502 12411292

[46] Gamadia LE Rentenaar RJ van Lier RA ten Berge IJ. Properties of CD4(+) T cells in human cytomegalovirus infection. Hum Immunol (2004) 65(5):486–92. doi: 10.1016/j.humimm.2004.02.020 15172448

[47] Lyu M Wang S Gao K Wang L Zhu X Liu Y. Dissecting the landscape of activated CMV-stimulated CD4+ T cells in humans by linking single-cell RNA-seq with T-cell receptor sequencing. Front Immunol (2021) 12:779961. doi: 10.3389/fimmu.2021.779961 34950144 PMC8691692

[48] Jung MK Shin EC. Regulatory T cells in hepatitis b and c virus infections. Immune Netw (2016) 16(6):330–6. doi: 10.4110/in.2016.16.6.330 PMC5195842 28035208

[49] Sakaguchi S Mikami N Wing JB Tanaka A Ichiyama K Ohkura N. Regulatory T cells and human disease. Annu Rev Immunol (2020) 38(1):541–66. doi: 10.1146/annurev-immunol-042718-041717 32017635

[50] Aandahl EM Michaëlsson J Moretto WJ Hecht FM Nixon DF. Human CD4+ CD25+ regulatory T cells control T-cell responses to human immunodeficiency virus and cytomegalovirus antigens. J Virol (2004) 78(5):2454–9. doi: 10.1128/JVI.78.5.2454-2459.2004 PMC369239 14963140

[51] Karlsson I Malleret B Brochard P Delache B Calvo J Le Grand R. FoxP3+ CD25+ CD8+ T-cell induction during primary simian immunodeficiency virus infection in cynomolgus macaques correlates with low CD4+ T-cell activation and high viral load. J Virol (2007) 81(24):13444–55. doi: 10.1128/JVI.01466-07 PMC2168878 17898053

[52] Diaz GA Koelle DM. Human CD4+ CD25 high cells suppress proliferative memory lymphocyte responses to herpes simplex virus type 2. J Virol (2006) 80(16):8271–3. doi: 10.1128/JVI.00656-06 PMC1563823 16873284

[53] Lorentino F Xue E Mastaglio S Giglio F Clerici D Farina F. Letermovir reduces chronic GVHD risk in calcineurin inhibitor-free GVHD prophylaxis after hematopoietic cell transplantation. Blood Adv (2022) 6(10):3053–7. doi: 10.1182/bloodadvances.2021006213 PMC9131907 35078219

[54] Abbas A Lichtman A Pillai S. Cellular and molecular immunology. In: Immunologic tolerance and autoimmunity, Elsevier, 9th Edition (2016). p. 334.

[55] Tey SK Kennedy GA Cromer D Davenport MP Walker S Jones LI. Clinical assessment of anti-viral CD8+ T cell immune monitoring using QuantiFERON-CMV(R) assay to identify high risk allogeneic hematopoietic stem cell transplant patients with CMV infection complications. PloS One (2013) 8(10):e74744. doi: 10.1371/journal.pone.0074744 24146744 PMC3795724

[56] Lee SM Kim YJ Yoo KH Sung KW Koo HH Kang ES. Clinical usefulness of monitoring cytomegalovirus-specific immunity by quantiferon-CMV in pediatric allogeneic hematopoietic stem cell transplantation recipients. Ann Lab Med (2017) 37(3):277–81. doi: 10.3343/alm.2017.37.3.277 PMC5339102 28224776

[57] Yong MK Cameron PU Slavin M Morrissey CO Bergin K Spencer A. Identifying cytomegalovirus complications using the quantiferon-CMV assay after allogeneic hematopoietic stem cell transplantation. J Infect Dis (2017) 215(11):1684–94. doi: 10.1093/infdis/jix192 28431019

[58] Chemaly RF El Haddad L Winston DJ Rowley SD Mulane KM Chandrasekar P. Cytomegalovirus (CMV) cell-mediated immunity and CMV infection after allogeneic hematopoietic cell transplantation: the REACT study. Clin Infect Dis (2020) 71(9):2365–74. doi: 10.1093/cid/ciz1210 PMC7713694 32076709

[59] Kay AW DiNardo AR Dlamini Q Kahari J Mndzebele T Mtetwa G. Evaluation of the QuantiFERON-tuberculosis gold plus assay in children with tuberculosis disease or following household exposure to tuberculosis. Am J Trop Med Hyg (2019) 100(3):540–3. doi: 10.4269/ajtmh.18-0674 PMC6402910 30675853

[60] ImmunoSpot. Available at: https://immunospot.com/media/mageplaza/product_attachments/attachment_file/c/t/ctl-cmv-kits-brochure.pdf (Accessed 12/01/2023).

[61] Lauruschkat CD Etter S Schnack E Ebel F Schäuble S Page L. Chronic occupational mold exposure drives expansion of aspergillus-reactive type 1 and type 2 T-helper cell responses. J Fungi (Basel) (2021) 7(9):698. doi: 10.3390/jof7090698 34575736 PMC8471116

[62] Lauruschkat CD Page L White PL Etter S Davies HE Duckers J. Development of a simple and robust whole blood assay with dual Co-stimulation to quantify the release of T-cellular signature cytokines in response to aspergillus fumigatus antigens. J Fungi (Basel) (2021) 7(6):462. doi: 10.3390/jof7060462 34201183 PMC8230040

[63] Lauruschkat CD Wurster S Page L Lazariotou M Dragan M Weis P. Susceptibility of a. fumigatus-specific T-cell assays to pre-analytic blood storage and PBMC cryopreservation greatly depends on readout platform and analytes. Mycoses (2018) 61(8):549–60. doi: 10.1111/myc.12780 29611226

[64] Dadwal SS Russo D Stelljes M Schmitt M Pilorge S Teal VL. 76 - A Phase 3 Randomized, Double-Blind, Placebo-Controlled Trial Evaluating the Safety and Efficacy of Letermovir (LET) Prophylaxis When Extended from 100 to 200 Days Post-Transplant in Cytomegalovirus (CMV)-Seropositive Recipients (R+) of an Allogeneic Hematopoietic Stem Cell Transplant (HSCT). Transplantation and Cellular Therapy (2023) 29(2, Supplement):S64. doi: 10.1016/S2666-6367(23)00145-8

[65] Dziedzic M Sadowska-Krawczenko I Styczynski J. Risk factors for cytomegalovirus infection after allogeneic hematopoietic cell transplantation in malignancies: proposal for classification. Anticancer Res (2017) 37(12):6551–6. doi: 10.21873/anticanres.12111 29187429

[66] Valadkhani B Kargar M Ashouri A Hadjibabaie M Gholami K Ghavamzadeh A. The risk factors for cytomegalovirus reactivation following stem cell transplantation. J Res Pharm Pract (2016) 5(1):63–9. doi: 10.4103/2279-042X.176554 PMC4776549 26985438

[67] Mallone R Mannering SI Brooks-Worrell BM Durinovic-Belló I Cilio CM Wong FS. Isolation and preservation of peripheral blood mononuclear cells for analysis of islet antigen-reactive T cell responses: position statement of the T-cell workshop committee of the immunology of diabetes society. Clin Exp Immunol (2011) 163(1):33–49. doi: 10.1111/j.1365-2249.2010.04272.x 20939860 PMC3010910

[68] Bull M Lee D Stucky J Chiu YL Rubin A Horton H. Defining blood processing parameters for optimal detection of cryopreserved antigen-specific responses for HIV vaccine trials. J Immunol Methods (2007) 322(1-2):57–69. doi: 10.1016/j.jim.2007.02.003 17382342 PMC1904432

[69] Shanaube K De Haas P Schaap A Moyo M Kosloff B Devendra A. Intra-assay reliability and robustness of QuantiFERON(R)-TB gold in-tube test in Zambia. Int J Tuberc Lung Dis (2010) 14(7):828–33. 20550764

