# Supplementary figures and images for: CD4+ T cells are the major predictor of HCMV control in allogeneic stem cell transplant recipients on letermovir prophylaxis
Source: Front Immunol. 2023 May 10;14:1148841. doi: 10.3389/fimmu.2023.1148841 (PMC10206124; doi:10.3389/fimmu.2023.1148841)

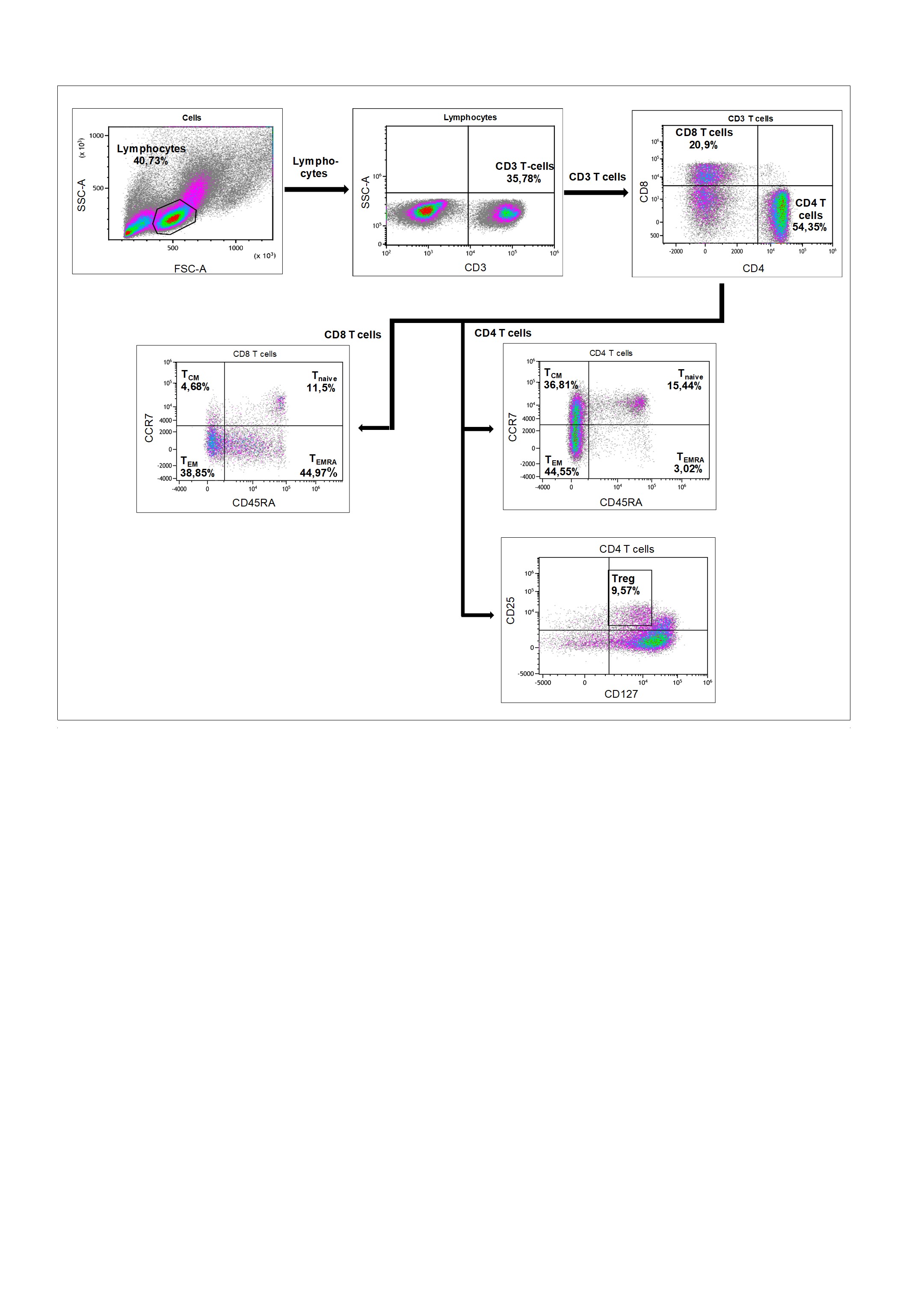

Supplement: Supplementary Figure 1 — Used gating strategy to study the T-cell phenotype. CD, cluster of differentiation, CM, central memory, EM, effector memory, EMRA, effector memory cells re-expressing CD45RA, Treg, regulatory T-cells. [file Image_1.jpeg]

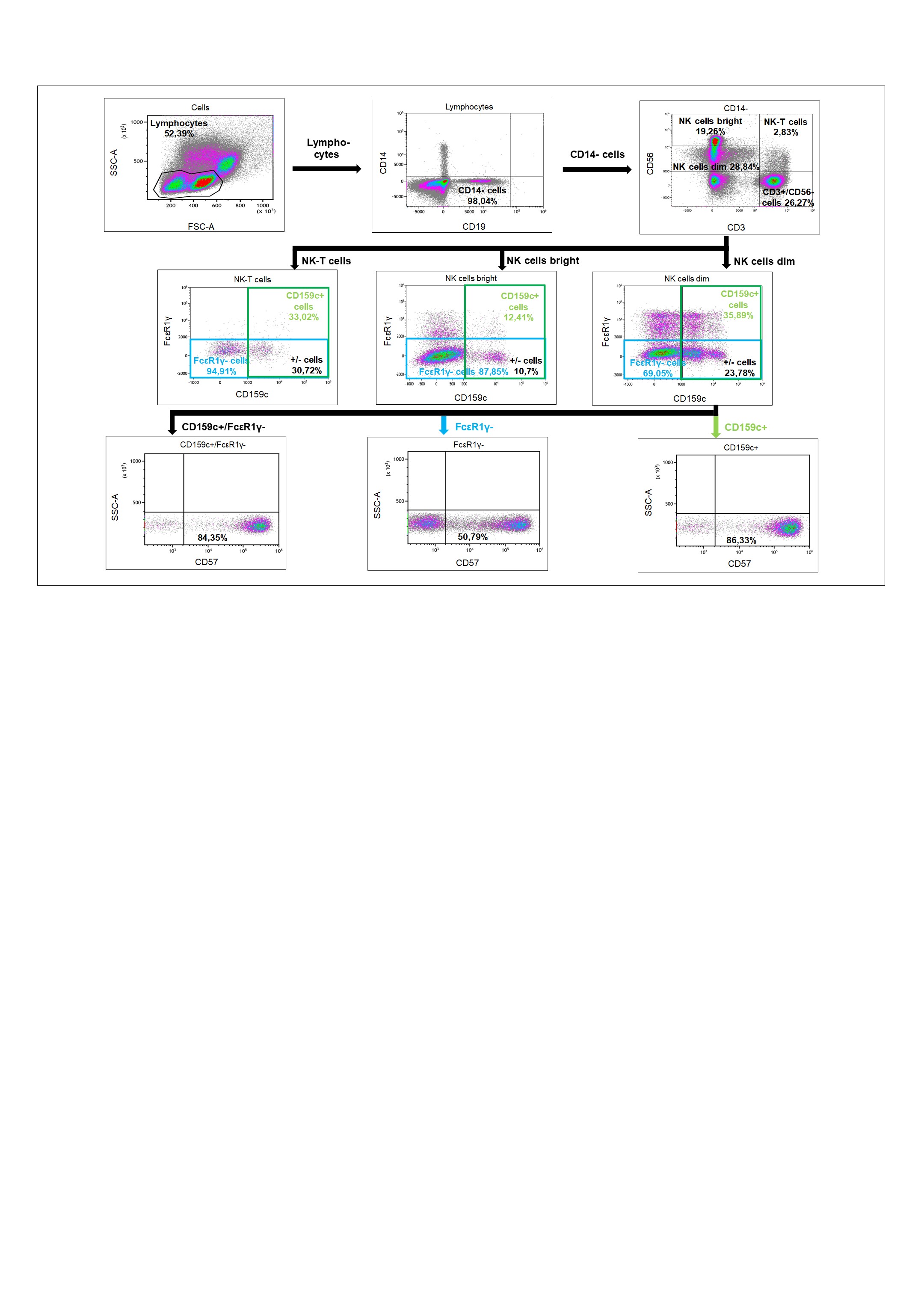

Supplement: Supplementary Figure 2 — Used gating strategy to study the NK-cell phenotype. CD, cluster of differentiation. [file Image_2.jpeg]

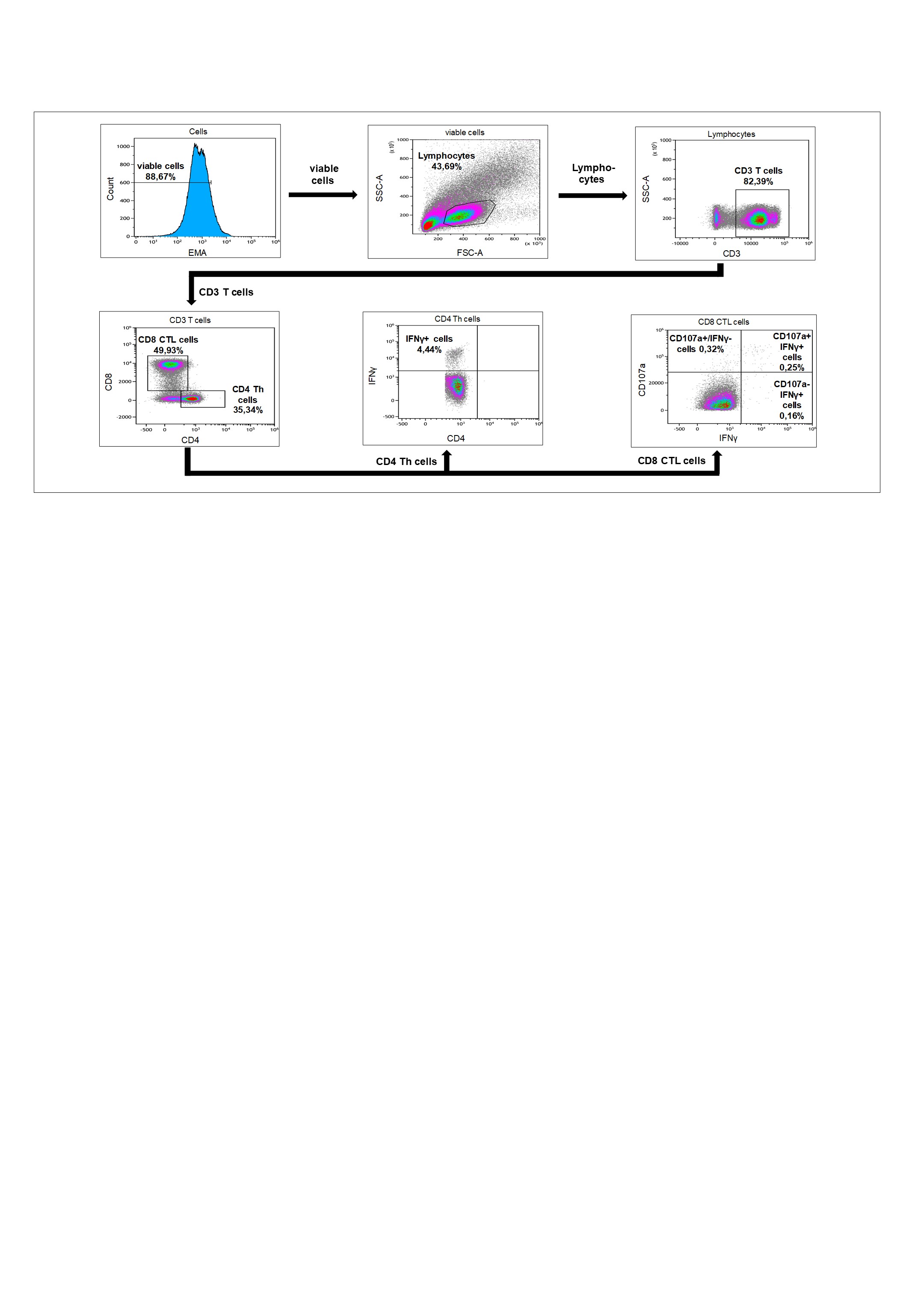

Supplement: Supplementary Figure 3 — Used gating strategy to study HCMV-specific T cells after stimulation. CD, cluster of differentiation. [file Image_3.jpeg]

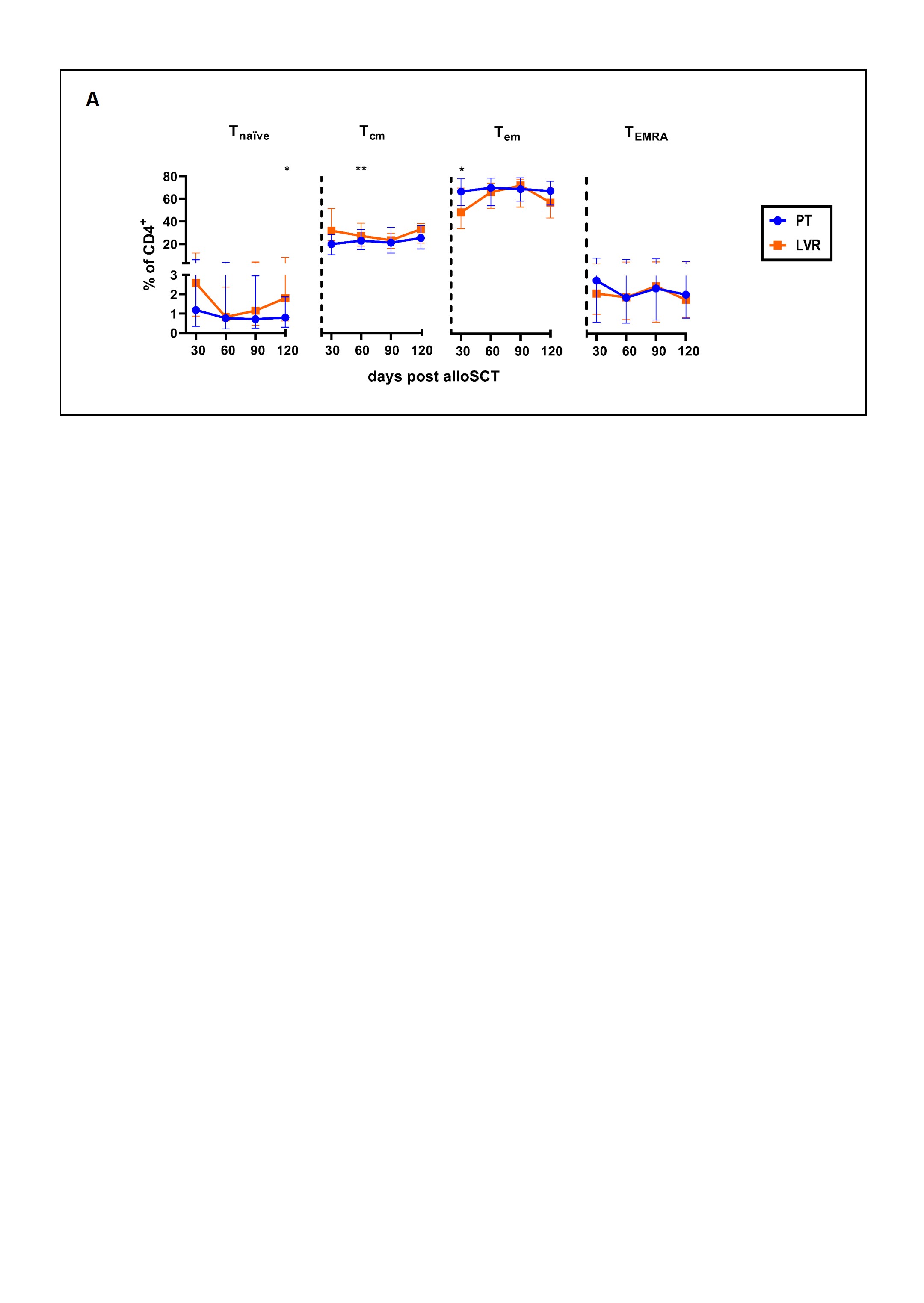

Supplement: Supplementary Figure 4 — Memory T-cell distribution in alloSCT recipients receiving letermovir prophylaxis or preemptive therapy. (A) Distribution of T-helper cells (CD4+): Tnaïve (CCR7+CD45RA+), Tcm (CCR7+CD45RA-), Tem (CCR7-CD45RA-), TEMRA (CCR7-CD45RA+). alloSCT, allogeneic stem cell transplantation, CD, cluster of differentiation, LVR, letermovir, PT, preemptive therapy, Tcm, central memory T cell, Tem, effector memory T cell, TEMRA, effector memory cells re-expressing CD45RA, Tnaïve, naïve T cells. [file Image_4.jpeg]
