# Supplementary material for: CD4+ T cells are the major predictor of HCMV control in allogeneic stem cell transplant recipients on letermovir prophylaxis
Source: Front Immunol. 2023 May 10;14:1148841. doi: 10.3389/fimmu.2023.1148841 (PMC10206124; doi:10.3389/fimmu.2023.1148841)
Supplement: Supplementary Table 1 — Patient characteristics of non/short-term and long-term HCMV reactivating patients after allogeneic stem cell transplantation receiving preemptive therapy. [file Table_1.pdf]

Table S1

Variables	Preemptive Therapy (n = 32)		
	**Non/short-term (n=18)**	**Long-term (n=14)**	***p****
**Age, median (range)**	52 (23 - 71)	57 (32 - 78)	0.35
**Sex, n (%)**			
Male	14 (78)	7 (50)	0.14
Female	4 (22)	7 (50)	
**Underlying disease, n (%)**			
Chronic leukemia	1 (6)	1 (7)	-
Multiple myeloma	3 (17)	0 (0)	
Acute leukemia	7 (39)	9 (64)	
Lymphoma	1 (6)	1 (7)	
Others	6 (33)	3 (20)	
**HLA matching, n (%)**			
Matched related	3 (17)	2 (14)	-
Matched unrelated	13 (72)	7 (50)	
Haploidentical	0 (0)	3 (21)	
Mismatch	2 (11)	2 (14)	
**Stem cell source, n (%)**			
PBSC	18 (100)	14 (100)	1
**Conditioning Regimen, n (%)**			
Reduced intensity	18 (100)	14 (100)	1
**Antithymocyte globulin, n (%)**			
No	0 (0)	3 (21)	0.07
Yes	18 (100)	11 (79)	
**HCT-CI, n (%)**			
0-2	10 (56)	9 (64)	-
3-4	5 (28)	3 (21)	
≥5	3 (17)	2 (14)	
**Serostatus (R/D), n (%)**			
+/+	17 (94)	10 (71)	0.14
+/-	1 (6)	4 (29)	
**aGvHD, n (%)**			
0-1	12 (67)	7 (50)	0.47
2-4	6 (33)	7 (50)	
**cGvHD, n (%)**			
No	13 (72)	14 (100)	0.05
Yes	5 (28)	0 (0)	
**Steroid AUC, median (range)**			
by day 100 (mg/kg per day)	0 (0 - 20)	5 (0 - 30)	0.09
**One-year mortality, n (%)**			
Alive	17 (94)	7 (50)	0.01*
Dead	1 (6)	7 (50)	

Table S2

**Variables**	**Letermovir (n = 24)**		
	**Non/short-term (n=17)**	**Long-term (n=7)**	***p****
**Age, median (range)**	63 (22 - 74)	52 (33 - 77)	0.91
**Sex, n (%)**			
Male	11 (65)	4 (57)	>0.99
Female	6 (35)	3 (43)	
**Underlying disease, n (%)**			
Chronic leukemia	0 (0)	1 (14)	-
Multiple myeloma	0 (0)	1 (14)	
Acute leukemia	11 (65)	5 (71)	
Lymphoma	1 (6)	0 (0)	
Others	5 (29)	0 (0)	
**HLA matching, n (%)**			
Matched related	3 (18)	2 (29)	-
Matched unrelated	12 (71)	3 (43)	
Haploidentical	0 (0)	0 (0)	
Mismatch	2 (12)	2 (29)	
**Stem cell source, n (%)**			
PBSC	17 (100)	7 (100)	1
**Conditioning Regimen, n (%)**			
Reduced intensity	17 (100)	7 (100)	1
**Antithymocyte globulin, n (%)**			
No	2 (12)	2 (29)	0.55
Yes	15 (88)	5 (71)	
**HCT-CI, n (%)**			
0-2	12 (71)	3 (43)	-
3-4	3 (18)	4 (57)	
≥5	2 (12)	0 (0)	
**Serostatus (R/D), n (%)**			
+/+	15 (88)	3 (43)	0.07
+/-	2 (12)	4 (57)	
**aGvHD, n (%)**			
0-1	12 (71)	5 (71)	>0.99
2-4	5 (29)	2 (29)	
**cGvHD, n (%)**			
No	15 (88)	5 (71)	0.55
Yes	2 (12)	2 (29)	
**Steroid AUC, median (range)**			
by day 100 (mg/kg per day)	0 (0 - 20)	0 (0 - 20)	0.42
**One-year mortality, n (%)**			
Alive	17 (100)	6 (86)	0.29
Dead	0 (0)	1 (14)	

Table S3

Fluorochrome		Panels		
		T-cell phenotpye	NK-cell phenotpye	HCMV-specific T cells
**VioBlue**	**Marker**		CD14	
	**Volume**		2 µl	
	**Company**		Miltenyi Biotec	
**V450**	**Marker**	CD8		CD8
	**Volume**	2 µl		2 µl
	**Company**	Becton Dickinson		Becton Dickinson
**V500**	**Marker**			CD4
	**Volume**			2 µl
	**Company**			Becton Dickinson
**BV510**	**Marker**		CD56	
	**Volume**		5 µl	
	**Company**		Biolegend	
**FITC**	**Marker**	CD3	FcεR1γ	IFNɣ
	**Volume**	5 µl	1 µl	5 µl
	**Company**	Becton Dickinson	Sigma-Aldrich	Beckman Coulter
**EMA**	**Marker**			L/D
	**Volume**			0.5 µg/ ml
	**Company**			Sigma-Aldrich
**PerCP-Cy5.5**	**Marker**	CD25		
	**Volume**	5 µl		
	**Company**	Becton Dickinson		
**ECD**	**Marker**	CD45RA	CD19	
	**Volume**	1 µl	5 µl	
	**Company**	Beckman Coulter	Beckman Coulter	
**PE**	**Marker**	CD197 (CCR7)		
	**Volume**	1 µl		
	**Company**	Miltenyi Biotec		
**PE-Cy7**	**Marker**	CD127		
	**Volume**	5 µl		
	**Company**	Biolegend		
**PE-Vio770**	**Marker**		CD159c	
	**Volume**		2 µl	
	**Company**		Miltenyi Biotec	
**APC**	**Marker**	CD4	CD57	CD107a
	**Volume**	1 µl	1 µl	2.5 µl
	**Company**	Becton Dickinson	Miltenyi Biotec	Becton Dickinson
**AF700**	**Marker**		CD3	CD3
	**Volume**		2 µl	2 µl
	**Company**		Becton Dickinson	Becton Dickinson

Table S4

day after alloSCT	d30				d60				d90				d120			
Figur/Treatment	PT		LVR		PT		LVR		PT		LVR		PT		LVR	
2A	32		21		29		23		31		23		32		24	
2B	32		21		29		23		31		23		32		24	
2C	32		21		29		23		31		23		32		23	
2D T cells	32		21		29		23		31		21		32		22	
2D NK cells	32		21		29		23		31		22		32		23	
3A	32		21		29		23		31		22		32		24	
3B	32		21		29		23		31		22		32		24	
3C both	32		21		29		23		31		22		32		24	
3D each	32		21		29		23		31		22		32		24	
3E	30		21		22		23		24		23		29		23	
3F	30		21		22		23		24		23		29		23	
4B each	32		21		29		23		31		23		32		23	
4C both	32		21		29		23		31		23		32		23	
4D both	32		21		29		23		31		23		32		23	
Reactivation	Non/short	Long	Non/short	Long	Non/short	Long	Non/short	Long	Non/short	Long	Non/short	Long	Non/short	Long	Non/short	Long
5B	18	14	15	6	17	12	16	7	18	13	17	5	18	14	17	7
5C	18	14	15	6	17	12	16	7	18	13	17	5	18	14	17	7
5D both	16	14	15	6	15	7	16	7	15	9	17	6	17	12	17	6
5E both	16	14	15	6	15	7	16	7	15	9	17	6	17	12	17	6
5F both	18	14	15	6	17	12	16	7	18	13	17	5	18	14	17	7
6A	18	14	15	6	17	12	16	7	18	13	17	6	18	14	16	7
6B	18	14	15	6	17	12	16	7	18	13	17	6	18	14	16	7
6C both	18	14	15	6	17	12	16	7	18	13	17	6	18	14	16	7
6D both	18	14	15	6	17	12	16	7	18	13	17	6	18	14	16	7
6E both	18	14	15	6	17	12	16	7	18	13	17	6	18	14	16	7
7B	–	–	–	–	–	–	16	7	–	–	–	–	–	–	–	–
7C	–	–	–	–	–	–	–	–	–	–	17	5	–	–	–	–
7D	–	–	–	–	–	–	16	7	–	–	–	–	–	–	–	–
7E	–	–	–	–	–	–	16	7	–	–	–	–	–	–	–	–
